# JNK pathway plays a key role in the immune system of the pea aphid and is regulated by microRNA-184

**DOI:** 10.1371/journal.ppat.1008627

**Published:** 2020-06-25

**Authors:** Li Ma, Lu Liu, Yujie Zhao, Lei Yang, Caihua Chen, Zhaofei Li, Zhiqiang Lu

**Affiliations:** 1 Department of Entomology, College of Plant Protection, Northwest A&F University, Yangling, Shaanxi, China; 2 State Key Laboratory of Crop Stress Biology for Arid Areas, Northwest A&F University, Yangling, Shaanxi, China; Pennsylvania State University, UNITED STATES

## Abstract

Different from holometabolous insects, the hemipteran species such as pea aphid *Acyrthosiphon pisum* exhibit reduced immune responses with the absence of the genes coding for antimicrobial peptide (AMP), immune deficiency (IMD), peptidoglycan recognition proteins (PGRPs), and other immune-related molecules. Prior studies have proved that phenoloxidase (PO)-mediated melanization, hemocyte-mediated phagocytosis, and reactive oxygen species (ROS) participate in pea aphid defense against bacterial infection. Also, the conserved signaling, Jun N-terminal kinase (JNK) pathway, has been suggested to be involved in pea aphid immune defense. However, the precise role of the JNK signaling, its interplay with other immune responses and its regulation in pea aphid are largely unknown. In this study, using *in vitro* biochemical assays and *in vivo* bioassays, we demonstrated that the JNK pathway regulated hemolymph PO activity, hydrogen peroxide concentration and hemocyte phagocytosis in bacteria infected pea aphids, suggesting that the JNK pathway plays a central role in regulating immune responses in pea aphid. We further revealed the JNK pathway is regulated by microRNA-184 in response to bacterial infection. It is possible that in common the JNK pathway plays a key role in immune system of hemipteran insects and microRNA-184 regulates the JNK pathway in animals.

## Author summary

As a model of hemipteran insects, pea aphid has recently been extensively studied. Interestingly, genomic analysis and biochemical assays revealed that some conserved core components, such as the immune deficiency pathway and peptidoglycan recognition proteins, are missing from the immune system of pea aphid. However, studies demonstrated that the phenoloxidase, reactive oxygen species, and phagocytosis play critical roles in the pea aphid defense against infection. Through bioinformatics analysis and biochemical assays, we found that these immune responses are under the control of the JNK pathway and that the latter is regulated by microRNA-184. Our results suggest that the JNK pathway is a key signaling pathway in the immune system of hemipteran insects, and the regulation of this pathway by microRNA-184 is conserved among animals.

## Introduction

Insects rely on physiological barriers and innate immune responses to defend themselves against pathogens and parasites. These immune responses have been described based on genetic, biochemical, and bioinformatic studies in the fruit fly *Drosophila melanogaster* [1, 2] and other insect species [3–8]. Generally, invading pathogens are recognized as non-self through interactions between pattern recognition receptors in the hosts and pathogen-associated molecular patterns present in pathogens, such as lipopolysaccharide, peptidoglycan, lipoteichoic acid, and β-1,3-glucans. The pattern recognition receptors include peptidoglycan recognition proteins (PGRPs), Gram-negative bacteria-binding proteins, scavenger receptors, thioester-containing proteins, and lectins [9, 10]. Upon recognition, signaling pathways such as Toll, immune deficiency (IMD), Jun N-terminal kinase (JNK), Janus kinase/signal transducers and activators of transcription (JAK/STAT), and prophenoloxidase (PPO) pathways are activated [1–6]. Activation of these pathways leads to defense responses, such as antimicrobial peptide (AMP) production, reactive oxygen species (ROS) generation, and melanization [1–8]. Hemocytes circulating in the blood participate in cellular responses, such as phagocytosis, encapsulation, and nodulation [11, 12].

Compared to holometabolous insects, the pea aphid *Acyrthosiphon pisum* exhibits reduced immune responses. Genomic data analysis suggested that the genes coding for PGRPs, scavenging receptor, IMD, AMPs, and other immune-related molecules are absent in the pea aphid [13]. Chromatography analysis and zone of inhibition assays revealed no detectable antimicrobial activity in the immune-challenged hemolymph [13, 14]. However, pea aphid exerts a hemocyte-mediated response, including phagocytosis and encapsulation, against bacteria and foreign intrusion [14, 15]. Recent studies from our and other groups showed that phenoloxidase (PO) is required and/or involved in the immune defense in pea aphid [16, 17]. Additionally, ROS play role in the interaction between pea aphid and bacteria [18, 19]. Expression profiling suggested that the JNK pathway is involved in the defense against invasive bacteria in pea aphid [20].

**Fig 1:**
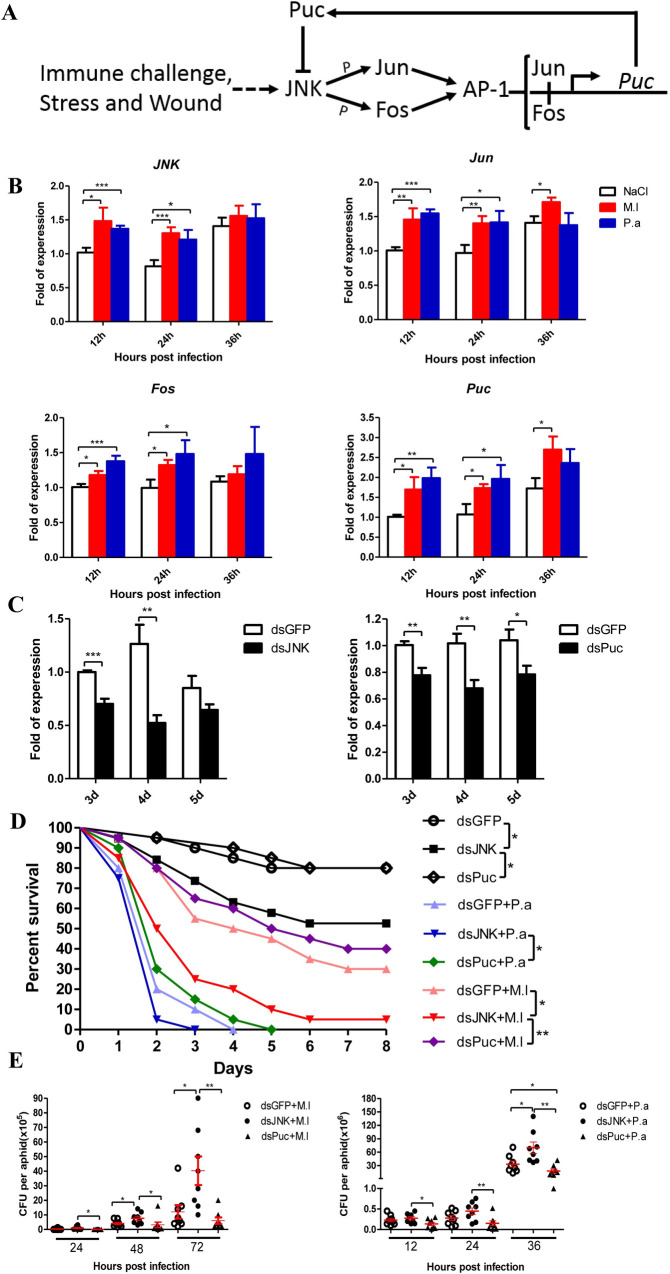
JNK pathway contributes to the pea aphid’s immune defense against bacterial infection. **(A)** The JNK signaling cascade of pea aphid based on functional studies from *Drosophila melanogaster* and *Anopheles gambiae*. **(B)** Relative expression levels of *JNK*, *Jun*, *Fos* and *Puc* in the pea aphids after Gram-positive bacteria *M*. *luteus* (M.l) and Gram-negative bacteria *P*. *aeruginosa* (P.a) infections with the aphids injected by sterile 0.85% as control groups. The expressions of *JNK*, *Jun*, *Fos* and *Puc* were normalized with ribosomal protein L7 gene (*rpl7*) of the pea aphids, and the relative expression of the infection groups were compared to the expression of the control groups at each time point. **(C)** Efficiency of RNA interference-mediated knockdown of the pea aphid *JNK* and *Puc*. The expressions of *JNK* and *Puc* were normalized with *rpl7* of the pea aphids, and the relative expression of the dsJNK and dsPuc injected groups were compared to the expression of the dsGFP groups at each time point. **(D)** Effect of *JNK* and *Puc* silence on the survival of the pea aphids after *M*. *luteus* (M.l) and *P*. *aeruginosa* (P.a) infection, n = 20. One representative survival graph from three independent experiments with similar results is shown. The statistical differences between the compared groups were denoted with asterisks. The log-rank (Mantel-Cox) test was used to analyze the pea aphids’ survival curves. *P<0.05; **P<0.01. **(E)** Effect of *JNK* and *Puc* silence on the bacteria loads of the pea aphids after *M*. *luteus* (M.l) and *P*. *aeruginosa* (P.a) infection, n = 8. Each dot in the graph represents an individual aphid. The horizontal bars indicate mean values and the vertical bars indicate the SEM of the replicates. The statistical differences between the compared groups were denoted with asterisks. P values were determined by Student’s *t* test. *P<0.05; **P<0.01. For (A) and (B), values shown are the mean (±SEM) of three independent experiments. The statistical differences between the control groups and infection groups were denoted with asterisks (A). The statistical differences between the dsGFP injected groups and dsJNK or dsPuc injected groups were denoted with asterisks (B). P values were determined by Student’s *t* test. *P<0.05; **P<0.01; ***P<0.001.

The JNK represents a subgroup of mitogen-activated protein kinases which are evolutionarily conserved in eukaryotic cells and activated by environmental stresses and inflammatory cytokines [21]. Upon activation, JNK phosphorylates the transcription factors Jun and Fos, leading to formation of the Jun/Fos dimer, i.e., AP-1 complex, which activates transcription of target genes. Puckered (puc), the product of a negative feedback loop, dephosphorylates JNK and suppresses the signaling (Fig 1A). In insects, exposure or injection of lipopolysaccharide triggers activation of the JNK pathway [22–24]. The antibacterial activity in the hemolymph of the greater wax moth *Galleria mellonella* larvae after lipopolysaccharide challenge was time- and dosage-dependent on JNK activation [24]. Further studies indicated that the JNK pathway is required for AMP gene expression in *Drosophila* [25, 26]. It was also found that the JNK pathway controls cytoskeletal gene expression and plays roles in cellular immune responses and wound healing [27–29]. JNK signaling is a key regulator in mosquito *Anopheles gambiae* limiting *Plasmodium* infection [30]. Additionally, the JNK pathway mediates the expression of enzymes that detoxify ROS and protects insect hosts from oxidative stress during infection [31–34]. These studies indicate that the JNK signaling plays role in insects’ defense against pathogens and parasites and promoted us to investigate its function and regulation in the pea aphid immune system.

MicroRNAs (miRNAs) are a class of endogenous non-coding RNAs generally 19–24 nucleotides in length. Typically, miRNA guides RNA-induced silencing complex to its target mRNA through binding to the 3′-untranslated region (UTR) and results in gene silencing by translational inhibition or mRNA degradation. Insect microRNAs function as regulators in many processes such as development, metamorphosis, immunity, and reproduction [35–38]. For instance, based on expression profiling and target prediction, microRNAs have been suggested to regulate gene expression in immune responses and pathways in the tobacco hornworm, honeybees, greater wax moth, and fruit fly [39–42]. Specifically, in fruit fly, microRNA-310 family members suppress the expression of Drosomysin, an AMP mediated by Toll signaling [43]; microRNA-317 directly targets the transcription factor *Dif-Rc* in the Toll pathway to down-regulate the expression of Drosomysin [44]. MicroRNA-9a and microRNA-981 target and repress the expression of AMP Diptericin, which is mediated by IMD signaling [45]; in contrast, microRNA-34 activates IMD signaling by repressing *Eip75B*, a negative regulator of the IMD pathway [46]. In the diamondback moth *Plutella xylostella*, the conserved microRNA-8 down-regulates activation of the Toll pathway and PPO cascade by up-regulating the serine protease inhibitor *Serpin 27*, which is a negative regulator of the Toll and PPO pathways [47]. In *A*. *gambiae*, microRNA-305 regulates the anti-plasmodium response possibly by targeting immune effector genes [48]. Certain microRNAs are also involved in insect-virus interactions [49, 50].

A total of 163 microRNAs have been identified in the pea aphid genome [51]. Sequencing and expression analysis indicated that some microRNAs are putative regulators involved in the switching of alternative morphs in pea aphid [52]. Recent studies identified a set of microRNAs that mediate the interaction between aphids and their obligate endosymbiont *Buchnera* [53, 54]. Little is known about role of microRNAs in the immune system of aphids to date. Previous studies demonstrated the critical roles of phagocytosis, PPO, and ROS in the pea aphid immune system [14–19] and suggested the involvement of JNK signaling in the aphid defense against bacteria [20]. However, the precise role of the JNK pathway and interplay between the JNK pathway and other immune responses in pea aphid remain unclear. In this study, we investigated the role of JNK pathway in the pea aphid immune system, how it regulates other immune responses, and its regulation by microRNA.

## Results

### JNK pathway was induced by bacterial infection and knockdown of JNK expression significantly affected aphid survival and bacterial load after infection

To determine whether the JNK pathway responds to microbial challenge in pea aphid, we first measured the expression of key genes in the JNK pathway (Fig 1A) by quantitative PCR after bacterial infection. Our results showed that *JNK*, *Jun*, *Fos*, and *Puc* were up-regulated after infection by *Micrococcus luteus* (Gram^+^) and *Pseudomonas aeruginosa* (Gram^-^) (Fig 1B). We knocked down the expression of *JNK* and *Puc* by RNA interference. Expression analysis showed that the mRNA levels of *JNK* and *Puc* were significantly decreased at 3–5 days after double-stranded RNA (dsRNA) injection (Fig 1C). Knockdown of JNK decreased *Puc* mRNA levels (S1 Fig), suggesting that the pea aphid JNK pathway regulates *Puc* expression. Knockdown of *JNK* resulted in higher mortalities of the aphids after *M*. *luteus* and *P*. *aeruginosa* infection; in contrast, knockdown of *Puc* resulted in lower mortalities (Fig 1D). Additionally, the propagation of bacterial cells inside aphids was examined. After infection, knockdown of *JNK* resulted in higher loads of *M*. *luteus* and *P*. *aeruginosa* inside the aphids; in contrast, knockdown of *Puc* led to lower bacterial loads (Fig 1E). Therefore, our results indicate that the JNK pathway contributes to the defense against bacterial infection in pea aphid.

### JNK pathway regulates ROS metabolism

**Fig 2:**
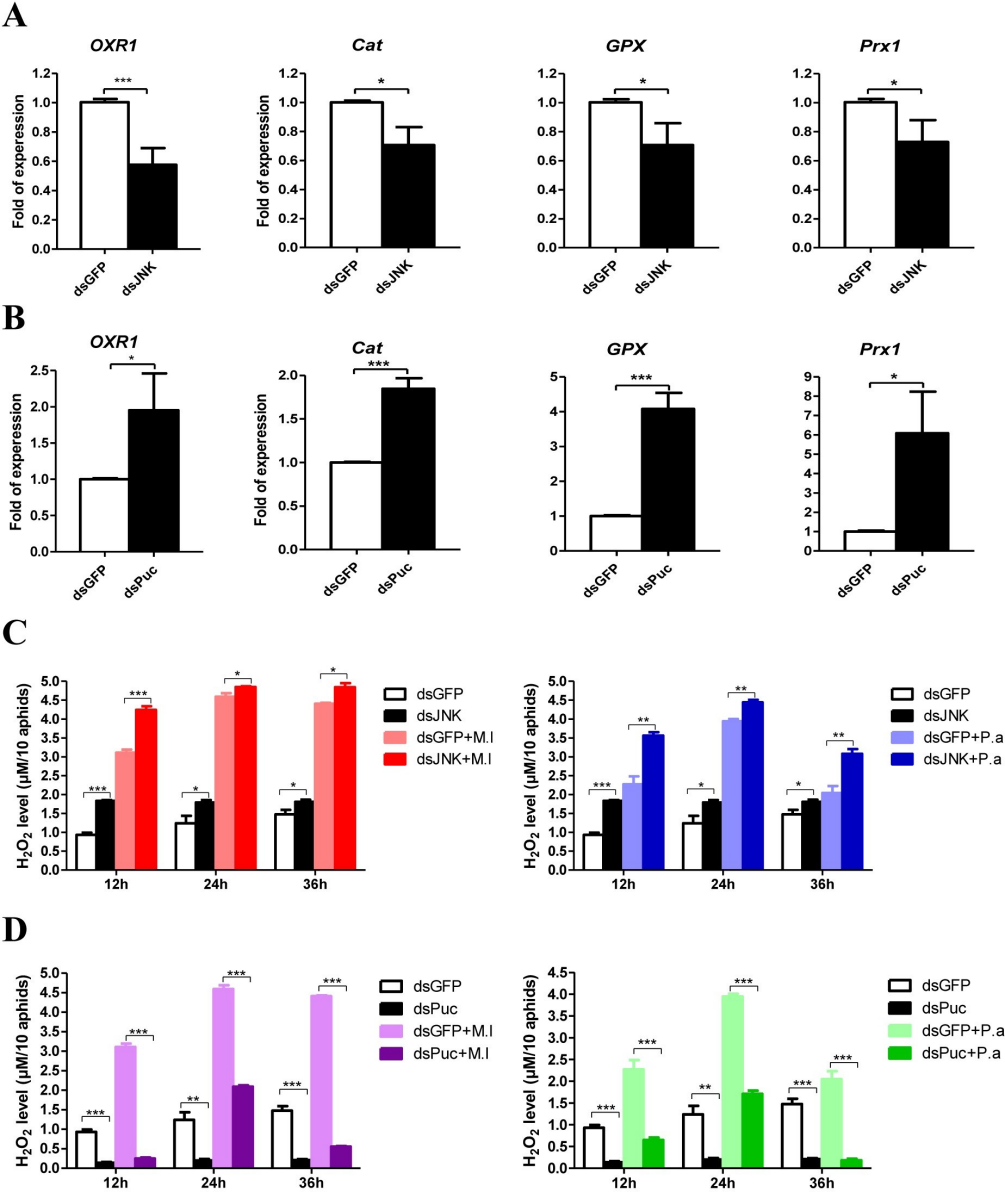
JNK pathway regulates ROS metabolism. Effect of *JNK* **(A)** and *Puc* **(B)** silence on the expression of antioxidant genes: *OXR1*, *Cat*, *GPX* and *Prx1* of the pea aphids. The expressions of *OXR1*, *Cat*, *GPX* and *Prx1* were normalized with *rpl7* of the pea aphids. The statistical differences between the dsGFP injected groups and dsJNK or dsPuc injected groups were denoted with asterisks. Effect of *JNK* **(C)** and *Puc* **(D)** silence on the H_2_O_2_ concentration in the aphids uninfected and infected by *M*. *luteus* (M.l) and *P*. *aeruginosa* (P.a). Ten aphids from each group at each time point were used for the measurements of H_2_O_2_ concentration. The statistical differences between the compared groups were denoted with asterisks. For **(A-D)**, the values shown are the mean (±SEM) of three independent experiments. P values were determined by Student’s *t* test. *P<0.05; **P<0.01; ***P<0.001.

Oxidation resistance 1 (OXR1) regulates expression of the ROS detoxification enzymes catalase (Cat) and glutathione peroxidase (GPX) through the JNK pathway in *A*. *gambiae* [33]. In insects, the activation mechanism of transcription factor activator protein (AP)-1 by the JNK pathway has been well-described [55]. AP-1 DNA binding sites were identified in the promoter regions of *OXR1* (S1 File: sequence 1). We investigated whether the pea aphid JNK pathway regulates the expression of ROS detoxification enzymes by regulating the expression of *OXR1*. Knockdown of *JNK* decreased *OXR1*, *Cat*, *GPX*, and *Prx1* (Peroxiredoxin 1) mRNA levels (Fig 2A), whereas silencing of *Puc* significantly increased the expression of these genes (Fig 2B). These results suggest that the pea aphid JNK pathway regulates ROS metabolism by controlling the expression of detoxification enzymes similarly as in *A*. *gambiae*. We further measured H_2_O_2_ concentrations in the aphids to confirm these results. After bacterial infection, the H_2_O_2_ level was increased significantly (Fig 2C and 2D). Under either uninfected or infected conditions, H_2_O_2_ levels were higher in *JNK* knockdown aphids than in aphids from the control groups (Fig 2C). In contrast, H_2_O_2_ levels were much lower in *Puc* knockdown aphids than in control group aphids (Fig 2D). Our results indicate that the JNK pathway mediates ROS homeostasis in pea aphid.

### JNK pathway regulates PPO pathway

**Fig 3:**
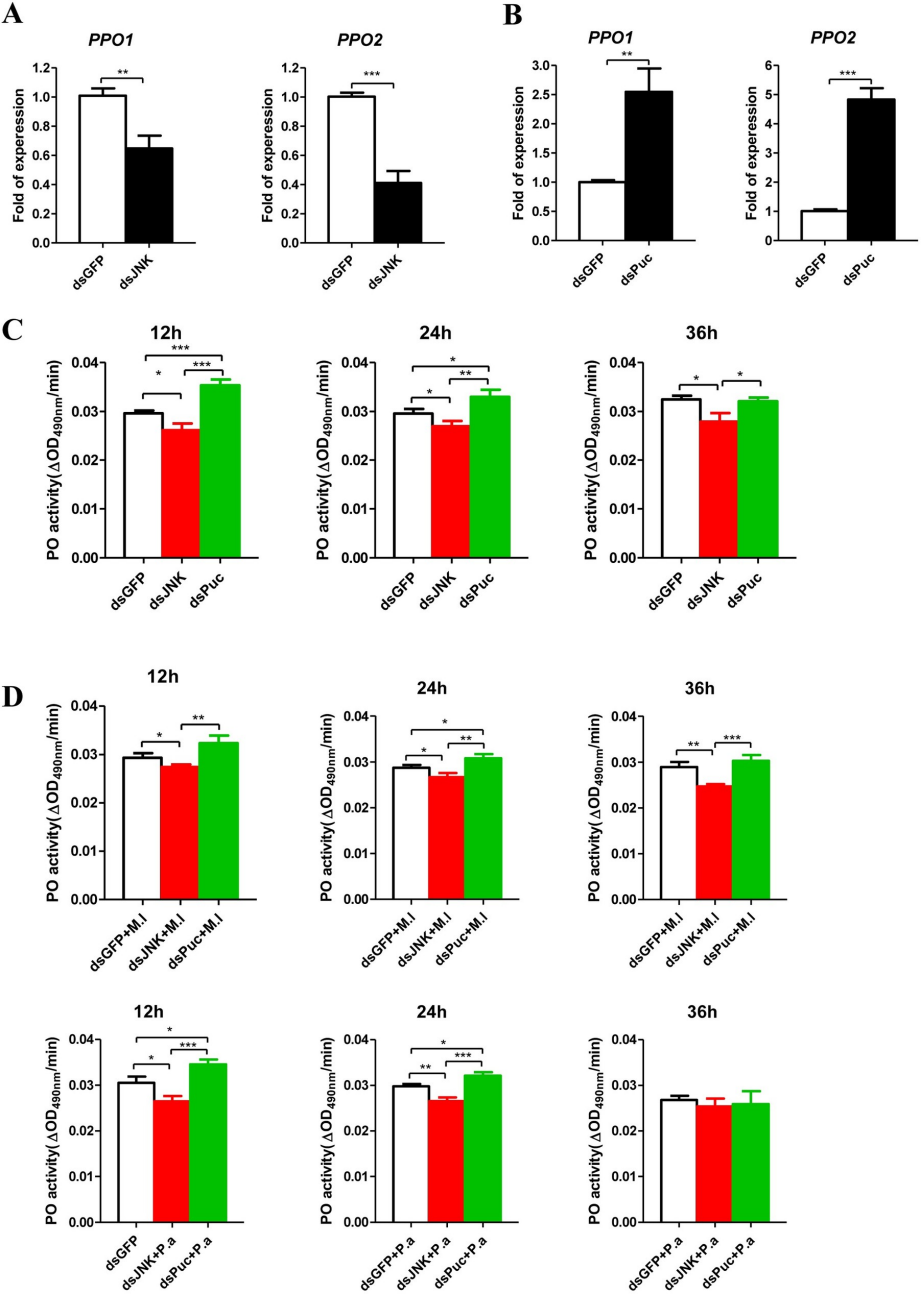
JNK pathway regulates PPOs expression and PO activity. Effect of *JNK* **(A)** and *Puc* **(B)** silence on the expression of *PPO1* and *PPO2* of pea aphids. The expressions of *PPO1*and *PPO2* were normalized with *rpl7* of the pea aphids. The statistical differences between the dsGFP injected groups and dsJNK or dsPuc injected groups were denoted with asterisks. **(C)** Effect of *JNK* and *Puc* silence on the PO activity in the uninfected aphids. **(D)** Effect of *JNK* and *Puc* silence on the PO activity in the aphids infected by *M*. *luteus* (M.l) and *P*. *aeruginosa* (P.a). For **(C)** and **(D)**, twenty aphids for the sample per group at each time point were used for measurements of PO activity. The statistical differences between the compared groups were denoted with asterisks. For **(A-D)**, the values shown are the mean (±SEM) of three independent experiments. P-values were determined by Student’s *t* test. *P<0.05; **P<0.01; ***P<0.001.

AP-1 has been shown to be a positive regulatory factor for the expression of *proPO* and melanization [56, 57]. AP-1 DNA binding sites were identified in the promoter regions of pea aphid *PPO1* and *PPO2* (S1 File: sequences 2 and 3). We next investigated whether the expression of *PPO* is regulated by the JNK pathway in pea aphid. Knockdown of *JNK* significantly decreased *PPO1* and *PPO2* mRNA levels (Fig 3A), whereas knockdown of *Puc* showed the opposite effects (Fig 3B). We next measured the PO activities in the aphid hemolymph after silencing of *JNK* and *Puc*. Under uninfected conditions, the knockdown of *JNK* decreased PO activity, whereas knockdown of *Puc* increased PO activity (Fig 3C). Following infection with *M*. *luteus* and *P*. *aeruginosa*, aphids in which *JNK* had been knocked down showed lower PO activity, whereas aphids with *Puc* knockdown showed higher activity (Fig 3D). These results suggest that the pea aphid JNK pathway regulates *PPO* expression and PO activity.

### JNK pathway mediates hemocytes phagocytosis

**Fig 4:**
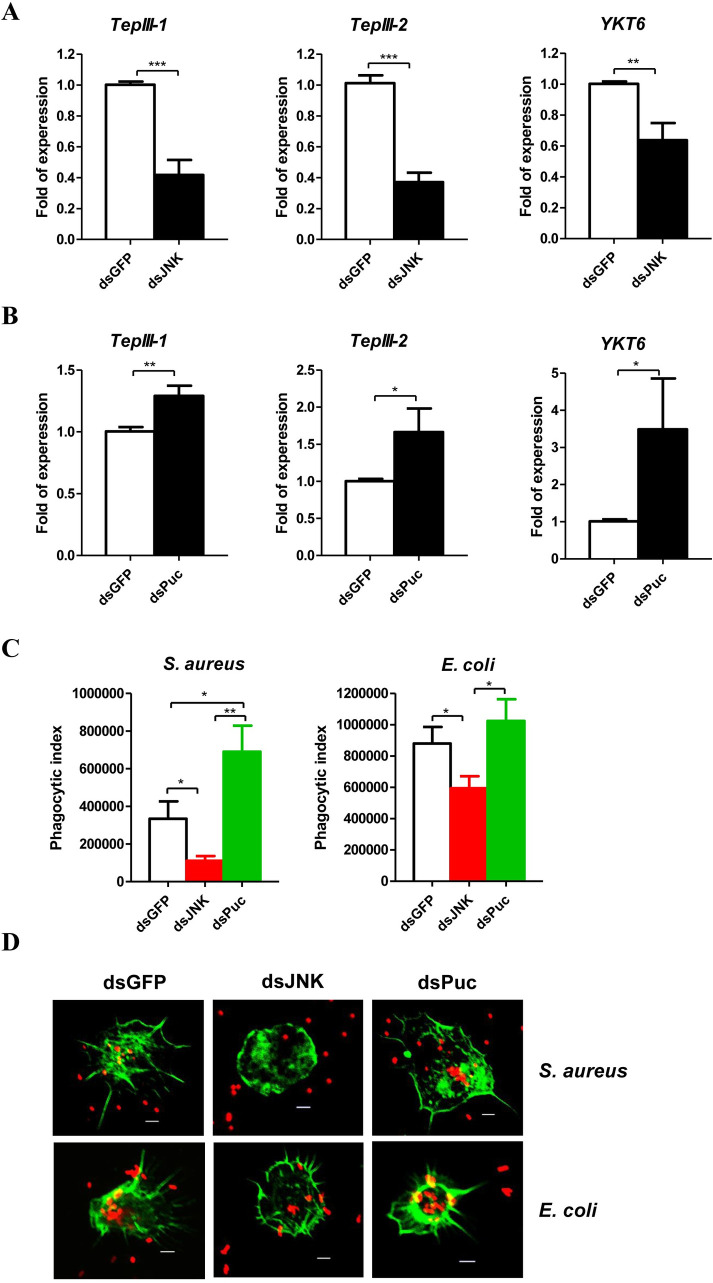
JNK pathway mediates hemocytes phagocytosis. Effect of *JNK* **(A)** and *Puc* **(B)** silence on the mRNA levels of phagocytosis related genes: *TepIII-1*, *TepIII-2* and *YKT6* of the pea aphids. The expressions of *TepIII-1*, *TepIII-2* and *YKT6* were normalized with *rpl7* of the pea aphids. **(C)** *Ex vivo* phagocytosis assay using *S*. *aureus* and *E*. *coli* AlexaFluor 594 BioParticle (Invitrogen) after knockdown of *JNK* and *Puc*. The hemocytes from 20 pea aphids per group were used to perform each experiment. In **(A-C)**, the values shown are the mean (±SEM) of three independent experiments and the statistical differences between the compared groups were denoted with asterisks. P-values were determined by Student’s *t* test. *P<0.05; **P<0.01; ***P<0.001. **(D)** The photographs of *ex vivo* phagocytosis *S*. *aureus* and *E*. *coli* AlexaFluo 594 BioParticles (Invitrogen) by the hemocytes with the F-actin stained by SF-488 Phalloidin (1/200 diluted, Solarbio) after knockdown of *JNK* and *Puc*. The red dots were *S*. *aureus* and *E*. *coli*, and the green parts were the hemocytes with the F-actin stained. Scale bar: 5 μm.

It has been reported that hemocytes phagocytosis contributes to the cellular immune response in pea aphid [15]. We predicted that the phagocytosis related genes *TepIII-1* and *TepIII-2* are regulated by the JNK pathway as in *Litopenaeus vannamei* [58]. AP-1 DNA binding sites were identified in the promoter regions of *TepIII-1*, *TepIII-2*, and *YKT6* (S1 File: sequences 4–6). Knockdown of *JNK* significantly decreased *TepIII-1*, *TepIII-2*, and *YKT6* mRNA levels (Fig 4A). In contrast, knockdown of *Puc* significantly increased the mRNA levels of these genes (Fig 4B). Additionally, the phagocytosis of bacteria by hemocytes after silencing of *JNK* and *Puc* was examined. Knockdown of *JNK* decreased phagocytosis, whereas knockdown of *Puc* showed the opposite effects (Fig 4C and 4D). Knockdown of *JNK* caused the hemocytes to have a rounded morphology (Fig 4D). These results clearly demonstrate that the JNK pathway mediates phagocytosis in pea aphid.

### miRNA-184 negatively regulates JNK pathway

**Fig 5:**
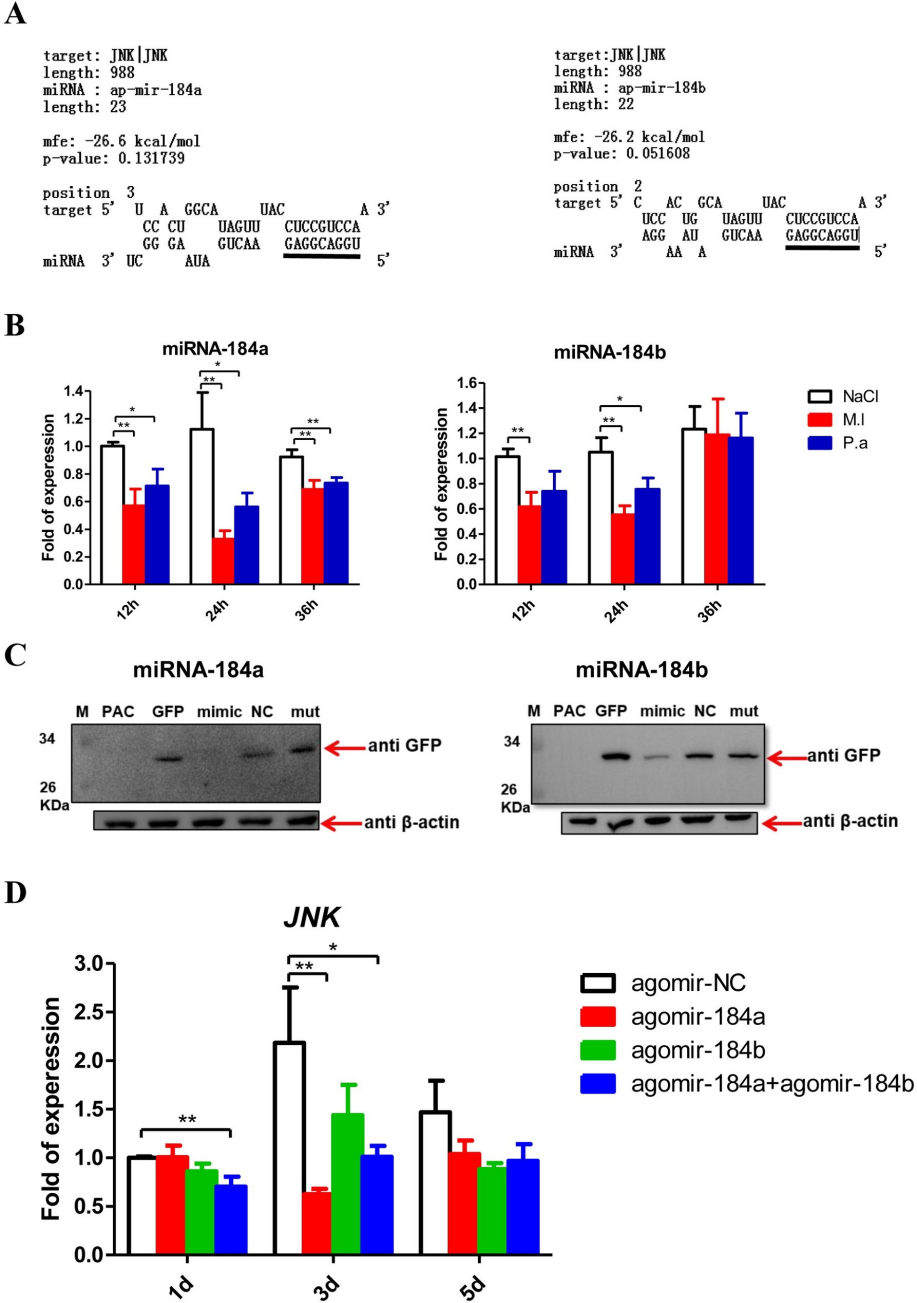
miR-184a/b target and negatively regulate JNK. **(A)** The putative miR-184a and miR-184b target binding site in 3′ UTR of *JNK* is predicted by using RNAhybrid. The sequences in the lines above were seed region (5′GGACGGA3′) binding sites predicted. **(B)** Relative expression levels of miR-184a and miR-184b in the pea aphids after *M*. *luteus* (M.l) and *P*. *aeruginosa* (P.a) infections with the aphids injected by sterile 0.85% as control groups. The expressions of miR-184a and miR-184b were normalized with U6 snRNA of the pea aphids, and the relative expression of the infection groups were compared to the expression of the control groups at each time point. **(C)** Western blotting of the GFP reporter assays showed that miR-184a and miR-184b directly degrade the 3′UTR of *JNK in vitro*. The upper arrows point to GFP reporter and the loading control β-actin was pointed by the nether arrows. M: Marker; PAC: pAc-5.1/V5-HisB plasmid was transfected into S2 cells alone, as mock or negative control; GFP: pAc-5.1/V5-HisB-GFP-JNK 3′UTR reporter plasmid was transfected into S2 cells alone, as positive control; mimic: pAc-5.1/V5-HisB-GFP-JNK 3′UTR reporter plasmid and mimic of miR-184a or miR-184b were co-transfected into S2 cells; NC: pAc-5.1/V5-HisB-GFP-JNK 3′UTR reporter plasmid and negative control mimic were co-transfected into S2 cells; mut: mutant mimic of miR-184a or miR-184b (at seed region: 5′-GGACGGA-3′ mutated as 5′-GACAUUC-3′) and pAc-5.1/V5-HisB-GFP-JNK 3′UTR reporter plasmid were co-transfected into S2 cells. **(D)** Relative expression levels of *JNK* in pea aphids after injected agomir-184a, agomir-184b and half-dose of agomir-184a plus half-dose of agomir-184b, with the aphids injected with agomir-NC as control group. The expressions of *JNK* were normalized with *rpl7* of the pea aphids, and the relative expression of the agomir-184 groups were compared to the expression of the control groups at each time point. For **(B)** and **(D)**, the values shown are the mean (±SEM) of three independent experiments and the statistical differences between the compared groups were denoted with asterisks. P values were determined by Student’s *t* test. *P<0.05; **P<0.01.

The targets of registered miRNAs in the pea aphid database were predicted *in silico*; the results showed that JNK is likely targeted by miRNA-184a and miRNA-184b at the *JNK*-3′UTR with the whole seed regions and low mfe value (Fig 5A). The expression of miRNA-184a and miRNA-184b remarkably declined after *M*. *luteus* and *P*. *aeruginosa* infection, with the lowest expression observed at 24 h post-infection (Fig 5B), revealing a negative correlation with *JNK* expression (Fig 1A). To confirm the interaction between miRNA-184a/b with *JNK* mRNA, a 739-bp DNA fragment of the *JNK*-3′UTR containing the target regions was cloned downstream of the *GFP* reporter open reading frame of pAc-5.1/V5-HisB vector (S4 Fig). After co-transfection into *Drosophila Schneider* S2 cells, mimics of miRNA-184a/b nearly completely abolished GFP expression, and miRNA-184a had a stronger effect than miRNA-184b (Fig 5C). Furthermore, *JNK* expression in aphids injected with agomir-184a, agomir-184b, and half-dose agomir-184a plus half-dose agomir-184b, with agomir-NC injected aphids as control group, was analyzed. The *JNK* mRNA levels decreased in the treated groups, with the effect produced by agomir-184a more obvious than by agomir-184b (Fig 5D). These results demonstrate that JNK is targeted and negatively regulated by miRNA-184a/b in pea aphid.

### miR-184 regulates ROS metabolism, PO activity, and hemocyte phagocytosis

**Fig 6:**
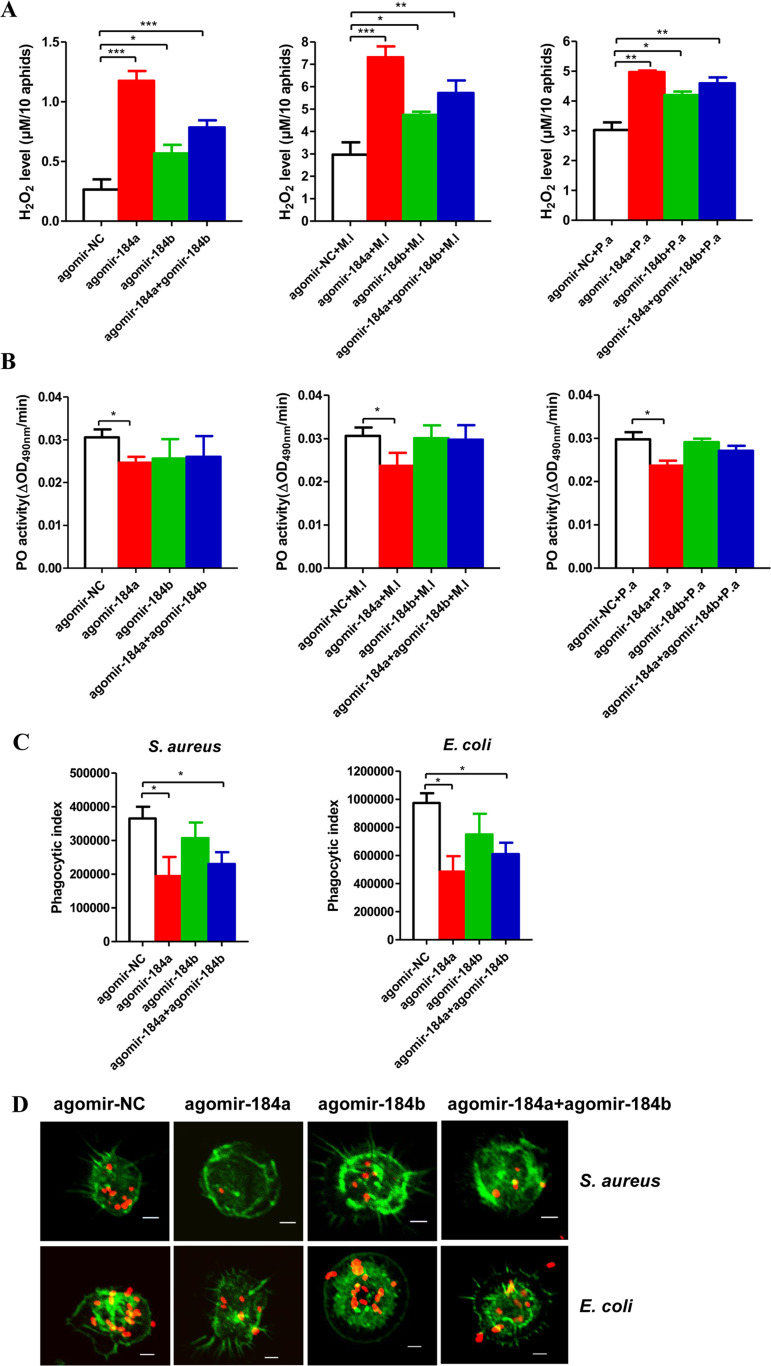
miR-184a/b regulate ROS metabolism, PO activity and hemocytes phagocytosis. Effect of injection of agomir-184a, agomir-184b and half-dose agomir-184a plus half-dose agomir-184b on H_2_O_2_ concentration **(A)** and the PO activity **(B)** in the aphids uninfected and infected by *M*. *luteus* (M.l) and *P*. *aeruginosa* (P.a) and the hemocytes phagocytosis **(C-D)**. Ten aphids for the sample per group at each time point were used for the measurements of H_2_O_2_ concentration. Twenty aphids for the sample per group at each time point were used for measurements of PO activity. The hemocytes from 20 pea aphids per group were used to perform each experiment. For **(A-C)**, the values shown are the mean (±SEM) of three independent experiments and the statistical differences between the compared groups were denoted with asterisks. P-values were determined by Student’s *t* test. *P<0.05; **P<0.01; ***P<0.001. **(D)** The photographs of *ex vivo* phagocytosis *S*. *aureus* and *E*. *coli* AlexaFluo 594 BioParticle (Invitrogen) by the hemocytes with the F-actin stained by SF-488 Phalloidin (1/200 diluted, Solarbio) after injection of agomir-184a, agomir-184b and half-dose agomir-184a plus half-dose agomir-184b. The red dots were *S*. *aureus* and *E*. *coli*, and the green parts were the hemocytes with the F-actin stained. Scale bar: 5 μm.

As described above, our results showed that the JNK pathway mediates ROS metabolism, PO activity, and hemocyte phagocytosis and that JNK is targeted and negatively regulated by miRNA-184a/b. Next, we assayed the H_2_O_2_ concentrations, PO activity, and hemocyte phagocytosis in pea aphids after agomir injection. Under both uninfected and infected conditions, the H_2_O_2_ levels were much higher in aphids injected with agomir-184a, agomir-184b, and half-dose agomir-184a plus half-dose agomir-184b than in the agomir-negative control (NC)-injected aphids (Fig 6A). PO activity was clearly lower in agomir-184a-injected aphids than in agomir-NC-injected aphids, whereas PO activity in agomir-184b- and half-dose agomir-184a plus half-dose agomir-184b-injected aphids showed a non-significant decrease compared to in agomir-NC-injected aphids (Fig 6B). In aphids injected with agomir-184, particularly agomir-184a, hemocytes exhibited reduced phagocytosis (Fig 6C and 6D). Therefore, miRNA-184a/b targets JNK signaling and regulates ROS generation, the PO pathway, and phagocytosis in pea aphids.

### miRNA-184 affects pea aphid survival and bacteria multiplication after infection

**Fig 7:**
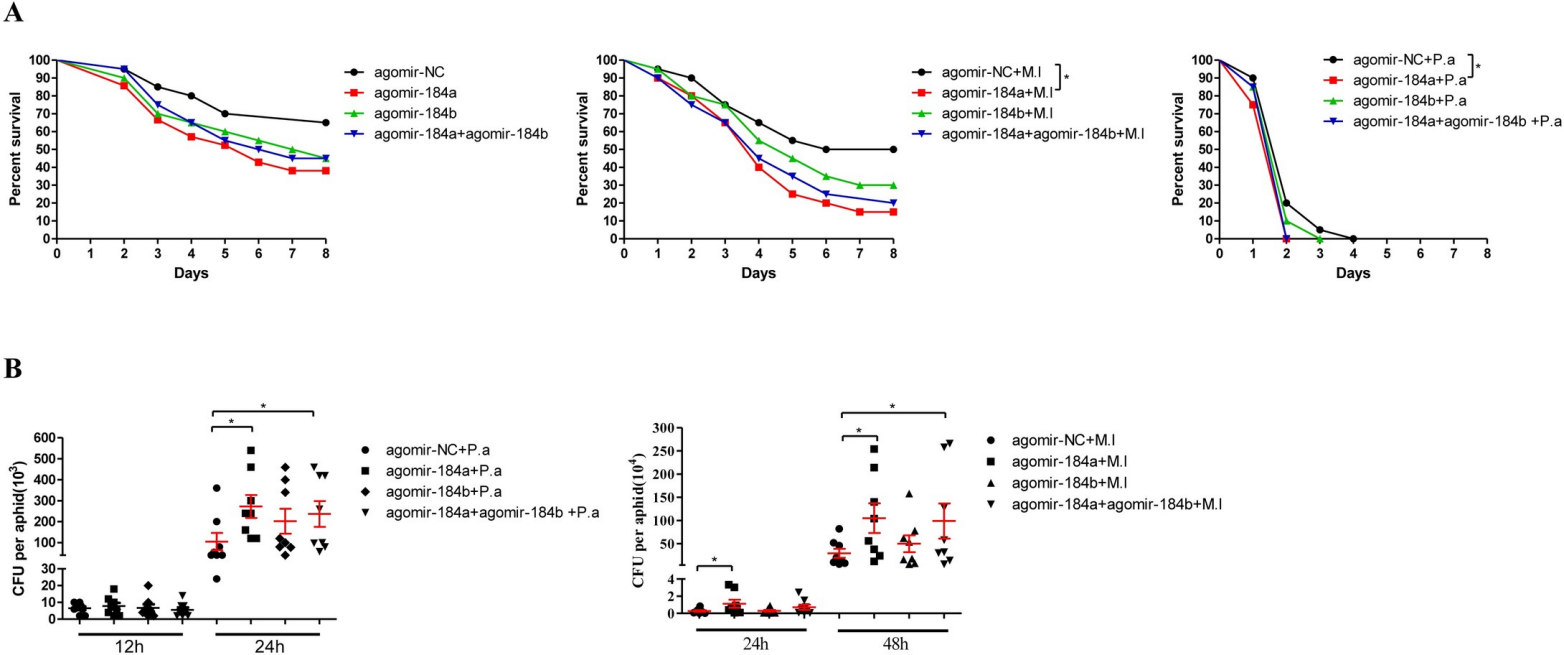
miRNA-184a/b affected pea aphid survival and bacteria multiplication after infection. **(A)** Effect of injection of agomir-184a, agomir-184b and half-dose agomir-184a plus half-dose agomir-184b on the survival of pea aphids after *M*. *luteus* (M.l) and *P*. *aeruginosa* (P.a) infection with the aphids injected by sterile 0.85% as control groups, n = 20. Survival graphs show one representative experiment out of three independent experiments with similar results. The statistical differences between the compared groups were denoted with asterisks. The log-rank (Mantel-Cox) test was used to analyze pea aphids’ survival curves. *P<0.05. **(B)** Effect of injection of agomir-184a, agomir-184b and half-dose agomir-184a plus half-dose agomir-184b on the bacterial load of pea aphids after *M*. *luteus* (M.l) and *P*. *aeruginosa* (P.a) infection, n = 8. Each dot in the graph represents an individual aphid. The horizontal bars indicate mean values and the vertical bars indicate the SEM of the replicates. The statistical differences between the compared groups were denoted with asterisks. P values were determined by Student’s *t* test. *P<0.05.

To confirm whether miRNA-184a and miRNA-184b affect the defense against bacterial infection in pea aphid, aphid survival and bacterial cell propagation in aphids were investigated. Under uninfected conditions, agomir-184a-, agomir-184b-, and half-dose agomir-184a plus half-dose agomir-184b-injected aphids showed higher mortalities compared to agomir-NC-injected aphids, although the difference was not significant (Fig 7A). Under *M*. *luteus*- and *P*. *aeruginosa*-infected conditions, agomir-184a-injected aphids showed obviously higher mortality than agomir-NC-injected aphids. Aphids injected with agomir-184b and half-dose agomir-184a plus half-dose agomir-184b showed higher mortality rates compared to agomir-NC injected aphids though the difference was not significant (Fig 7A). In infected aphids, injection with agomir-184a and half-dose agomir-184a plus half-dose agomir-184b resulted in remarkably higher loads of *M*. *luteus* and *P*. *aeruginosa* compared to after agomir-NC injection (Fig 7B). Together, these results suggest that miRNA-184a/b negatively regulates pea aphid immune responses, resulting more bacteria in the aphids and more aphids’ death after infection.

## Discussion

**Fig 8:**
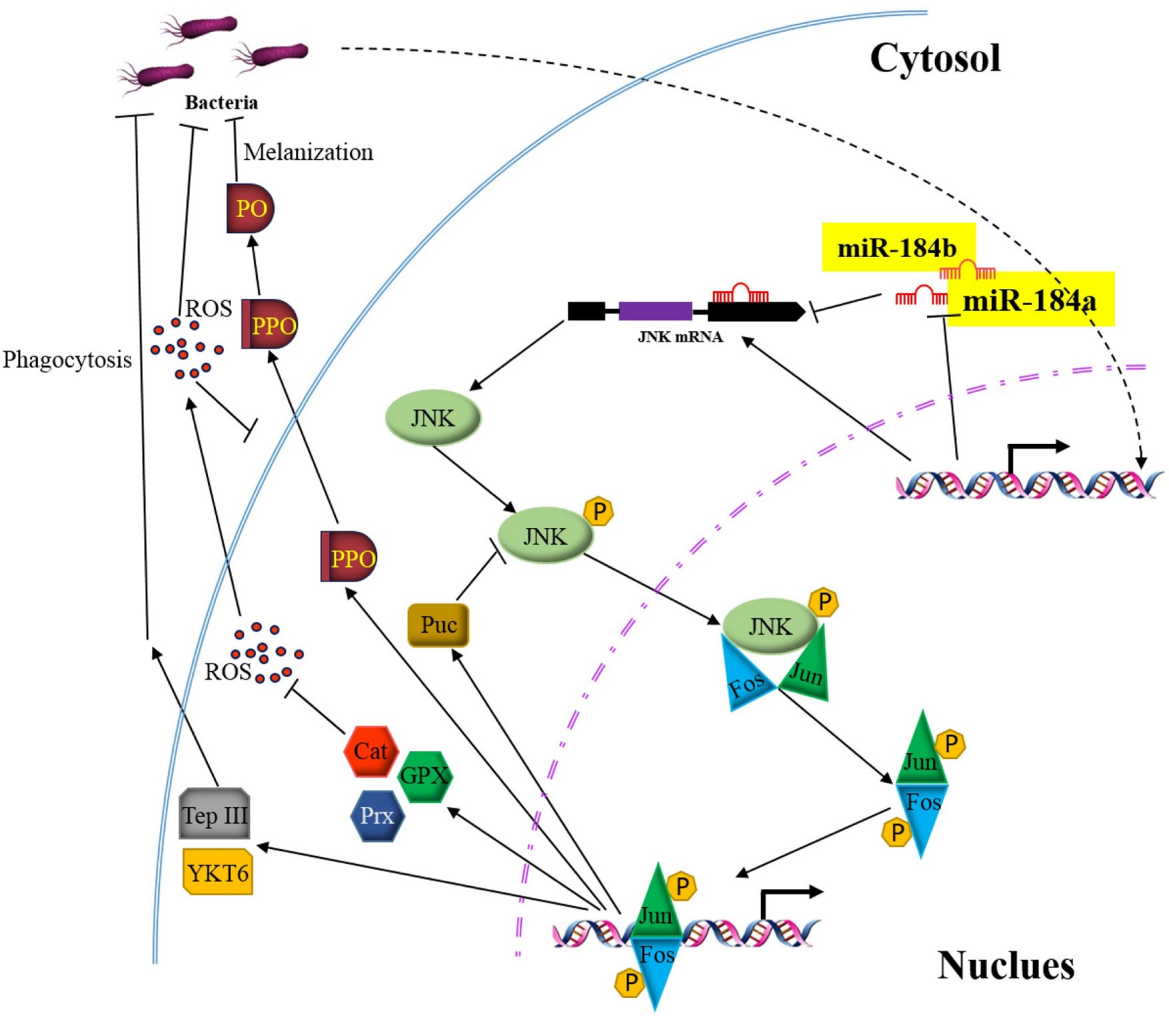
A schematic summary of the role of JNK pathway that modulates pea aphid immune signaling and is negatively regulated by miR-184. The transcription factor AP-1 is activated through Jun and Fos that are phosphorylated by JNK. AP-1 upregulates PO activity, increases hemocytes phagocytosis, and upregulates genes expression for ROS detoxification. JNK expression is negatively regulated by miRNA-184a/b. Bacterial infection downregulates synthesis of miRNA-184, leading to deliverance of JNK pathway and activation of immune responses consequently, to protect pea aphid from infection and oxidative stress.

JNK signaling mediates and regulates diverse processes in eukaryotic cells in response to abiotic and biotic stresses [59]. In insects, the JNK pathway is involved in immune responses, wound healing, and oxidative homeostasis [22–34]. In this study, we demonstrated that the JNK pathway mediates and controls phagocytosis, PPO activation, and ROS metabolism in pea aphids after bacterial infection. The JNK pathway is under control by miRNA-184 and is liberated after infection (summarized in Fig 8). Considering that pea aphids lack pathogen-associated molecular pattern recognition proteins (for instance, peptidoglycan recognition proteins) and complete IMD signaling and contain detectable antimicrobial peptides, our findings highlight the central role of JNK signaling in the immune system of aphids.

It has been known that the secondary symbiotic bacteria protect aphids from wasp attack and fungal infection [60–62]. The abundance and community structure of symbiotic bacteria is profoundly affected by the ROS level of their hosts [63–66]. On the other hand, symbiotic bacteria are able to regulate the host immune pathways through induction of ROS [67]. In the mosquito *Aedes aegypti*, knockdown of *JNK* reduced ROS level and increased microbiome load in the gut [68]. Conversely, knockdown of *JNK* resulted in an increase of ROS level in our study (Fig 2C). At this moment we do not know how the pathogen, symbiotic bacteria and aphid interact and how the interaction is modulated to maintain homeostasis. ROS is likely the key player in the interaction.

Generally, JNK activation relies on Eiger-Wengen or IMD pathways in insects [69]. This indicates that the JNK pathway and IMD pathway are closely related functionally. The absence of IMD canonical components is common in hemipteran species including pea aphid [70]. However, the JNK pathway is retained in these insects (S3 Fig). Interestingly, Wengen is missing in all five hemipteran species we examined (S3 Fig). This suggests other possible JNK activation mechanisms, such as by platelet-derived and vascular endothelial growth factor receptor, platelet-derived growth factor/vascular endothelial growth factor receptor, or Alk [3]. Overall, our results indicate that the JNK pathway plays a critical role in hemipteran insects.

We further revealed the JNK pathway is regulated by miRNA-184a/b in pea aphid. miRNA-184 is highly conserved in animals (S5 Fig). Several studies have suggested that miRNA-184 is involved in the proliferation and survival of cancer cells as a regulator [71–74]. In umbilical cord blood-derived CD4^+^ T cells, microRNA-184 inhibits nuclear factor of activated T cells-1 and plays a role in the early adaptive immune response [75]. miRNA-184 is also involved in female germline development [76], peripheral nervous system development [77], and metabolism and aging [78] in *Drosophila*. Large-scale screening revealed that miRNA-184 expression was down-regulated after *Escherichia coli* and *M*. *luteus* infection [43 and 45]. We demonstrated in this study that miRNA-184a/b expression was down-regulated in pea aphids after *P*. *aeruginosa* and *M*. *luteus* infection. Through bioinformatics prediction and *in vitro* and *in vivo* assays, we found that miRNA-184 negatively regulates JNK signaling in pea aphid. Prediction using the RNAhybrid program showed that JNK is a potential target of miRNA-184 in insects, zebrafish, frog, mouse, and human (S2 File). Therefore, the regulation of the JNK pathway by miRNA-184 is likely a universal mechanism in animals.

## Materials and methods

### Aphid rearing

The *A*. *pisum* strain was originally collected from Yunnan, China and derived from a single parthenogenetic female. The aphid colonies were maintained on broad bean (*Vicia faba*) seedlings in a growth chamber at 21 ± 1°C and 70 ± 5% relative humidity under a 16-h light (L): 8-h dark (D) photoperiod. Ten adult female aphids were placed on each seedling and allowed to produce offspring for two days. The adults were then removed from the seedlings. The nymphs were reared on the plants until they reached wingless adults. These newly emerged aphid adults were used in the following experiments.

### Bacterial infection

Gram-negative bacteria *P*. *aeruginosa* (PAO1, from Dr. Xihui Shen at Northwest A&F University) and Gram-positive bacteria *M*. *luteus* were cultured in Luria-Bertani liquid medium at 37°C and their growth was monitored by measuring the absorbance of the culture at 600 nm until the optical density reached approximately 1. The cells were then harvested by centrifugation and the pellets were resuspended in sterilized 0.85% NaCl solution to bring the final *P*. *aeruginosa* cell suspension to 2 × 10^9^ colony formation units (CFU)/mL and *M*. *luteus* cell suspension to 2 × 10^10^ CFU/mL. The adult aphids were anesthetized on ice and pricked with a sterile capillary dipped into bacteria suspensions or sterilized 0.85% NaCl solution as previously described [18].

### Agomir and dsRNA injection

Agomir-184a/b and agomir-NC, which are chemically modified double-strand stable mimics, were synthesized by GenePharma (Shanghai, China). The target fragments for RNA interference were amplified by PCR using the primers listed in Table 1. The PCR products were purified using a Gel Extraction Kit (Omega, Norcross, GA, USA) and then used to synthesize dsRNA with the T7 RiboMAX Express RNAi System (Promega, Madison, WI, USA) according to the manufacturer’s instructions. dsRNA was quantified by spectrophotometric analysis, and purity and integrity were verified by agarose gel electrophoresis.

**Table 1:** Primers used in the study.

Primers	sequence (5'-3')
**Quantitative RT-PCR** *JNK* F *JNK* R *Jun* F *Jun* R *Fos* F *Fos* R *Puc* F *Puc* R *Eiger* F *Eiger R* *ORX1* F *ORX1* R *PPO1* F *PPO1* R *PPO2* F *PPO2* R *TepIII-1* F *TepIII-1* R *TepIII-2* F *TepIII-2* R *YKT6*-F *YKT6-*R *Catalase* F *Catalase* R *GPX* F *GPX* R *Prx1* F *Prx1* R *Rpl7* F *Rpl7* R miR-184a F miR-184a R miR-184b F miR-184b R *U6* F *U6* R **dsRNA synthesis** ds*JNK* F ds*JNK* R ds*Puc* F ds*Puc* R ds*Eiger* F ds*Eiger* R ds*GFP* F ds*GFP* R **Recombinant transfection plasmid** *GFP* F *GFP* R *JNK*-3’UTR F *JNK*-3’UTR R	GACCATGGGACCATTCAGTAG CTGCTGCAGTAGTTGGATCATA CCGTCTCTATTTCCCAAG CAACAACGATTCTAACAGTGCC GGTCCGAAGAGAACGTAACAA CTAACTGCCCAGTTTCCTCTAA GAGGCGTGGTTACAGAGAAA GTCCACTAACGATGCAAGGA GGAGATCGATTTATGGGCAGAG GTTGCATCAATCACTGCGTTC TTACGCGACCAGCGTTAAT AGCTGTTTCCTGTGGTCTTC GCTATTGTGGTATTCGTAA CTGTTGGCTTCCTATTCTGT CACTGTCCGTAGCATTGAT GGCAGAATAATCGTGAGGTA CTCGAGGTTCAGGTGGTTTAG CCCGGCTTATAGATGGCTTTAT GACGGATCGCCATTAGATAGATAC GTTCTTCGTCCGCCAGATT TTACCACCAATATGACGACTGG GGGAAGGGATCGGAATGTAATAA CCTGTAAATTGTCCGTATCG AGTGAGGTTGCTGTTCTG CAAATCGTCGGAGGAGGATAAC CGCGTAATTCACAACGATCAAC AAAATTCAAGGGCACTGCTG CTGAAGTGGCTATCGCATGA TTGAAGAGCGTAAGGGAACTG TATTGGTGATTGGAATGCGTTG TGCCAGAACTGATAAGGGCT universal primer in miScript SYBR Green PCR Kit TGCCAGAACTGATAAAGGA universal primer in miScript SYBR Green PCR Kit CGATACAGAGAAGATTAGCATGG GTGGAACGCTTCACGATTTT *ACTATTACTCAACAAAGTGTTG *CAGGAAACAATACGCCACCT *TGTCGGCTGTCTGTTACGAG *GACGTGCCATTTTGTTGATG *ATGTGCTCTGCTACGTCCCA *CTGCCCATAAATCGATCTCC *GTGTTCAATGCTTTTCCCGT *CAATGTTGTGGCGAATTTTG CTTGGTACCATGGCTAGCAAAGG CCAGTGGAGTTTATTTGTAGAGCTCATC CTACAAATAAACTCCACTGGCATAGTTT CCTGGAATTCTTACAGACCTAAGAATG

Underline showed the *Kpn* I and *EcoR* I restriction enzyme sites.

* Only gene-specific parts of the primers are listed. These are preceded by the T7 adaptor TAATACGACTCACTATAGGG for dsRNA synthesis.

Newly emerged adult pea aphids were anesthetized with CO_2_ and injected with 50 nL of agomir (0.4 pmol/nL) or dsRNA (10,000 ng/μL) at the dorsal site of the abdomen using a Nanoject III micro-injector (Drummond Scientific, Broomall, PA, USA) equipped with glass capillaries prepared using a P-97 Micropipette Puller (Sutter Instrument Co., Novato, CA, USA). Agomir-NC and dsGFP were injected as controls. After injection, the pea aphids were transferred to fresh broad bean seedlings.

### RNA extraction, cDNA synthesis, and quantitative real-time PCR (qRT-PCR)

Total RNA containing small RNA was extracted and purified by using the High Pure miRNA Isolation Kit (Roche, Basel, Switzerland) according to the manufacturer’s instructions. cDNA for miRNAs was reverse-transcribed from 1 μg of extracted total RNA using the miScript II RT Kit (Qiagen, Hilden, Germany), in which HiFlex Buffer was used so that miRNA and mRNA could be quantified in parallel. qRT-PCR for mature miRNA was conducted using the miScript SYBR Green PCR Kit (Qiagen) according to the manufacturer’s protocol. For mRNA quantitation, qRT-PCR was performed by using Faststart Essential DNA Green Master (Roche). The U6 snRNA and ribosomal protein L7 (*rpl7*) genes were used as endogenous controls for miRNAs and mRNAs, respectively. The results were evaluated using a relative quantitative method (2^-ΔΔCt^). All analyses were performed with three biological replicates. The primers used in qRT-PCR are listed in Table 1. The R^2^ values of the standard curves were over 0.980 and the calculated amplification efficiency was 90% ~110%. These indicated that the qRT-PCR reactions were done in optimal condition.

### Aphids survival recording and bacterial CFUs counting

Two days after agomir injection or three days after dsRNA injection, the pea aphids were infected with bacteria as described above. Twenty pea aphids in each group were to analyze survival for 8 days at one-day intervals.

To determine the bacterial CFU, the pea aphids were surface-sterilized with 75% ethanol and then washed with 0.85% NaCl solution to remove residual ethanol. Each aphid was ruptured in sterile 0.85% NaCl solution. After diluting to a suitable concentration that are easy for counting of the colonies on the plates, the ruptured mixture was evenly spread onto Luria-Bertani agar plates, and bacterial colonies were counted after culture at 37°C overnight.

### Phenoloxidase activity assay

For each group, 20 decapitated pea aphids were placed in a 0.5-mL Eppendorf tube with a filter of sterile degreasing cotton that had been inserted into a 1.5-mL Eppendorf tube and centrifuged at 500 ×*g* for 10 min in a 4°C to collect the hemolymph. Two microliters of hemolymph and 100 μL L-dopamine (2 mmol/L in 50 mmol/L Tris-HCl, pH 8.0) were promptly mixed in each well of a 96-well plate, and the absorbance at 490 nm was immediately measured on a microplate reader (Tecan, Männedorf, Switzerland). Absorbance was measured every 30 s for 30 min. PO activity was detected as the maximum slope, which was defined as an increase in absorbance at 490 nm/min [79]. Three independent biological replicates were evaluated for each treatment.

### H_2_O_2_ measurement

The whole body H_2_O_2_ concentration in pea aphid was determined as described previously [19 and 67]. The pea aphids were collected in 50 mM sodium phosphate buffer (pH 7.4) containing 2 mg/mL catalase inhibitor 3-amino-1, 2, 4-trizole (Sigma, St. Louis, MO, USA). After homogenization, the samples were filtered through a 10-kDa molecular weight cutoff spin filter (Millipore, Billeeica, MA, USA). The eluent was collected and the H_2_O_2_ concentration was measured with a Hydrogen Peroxide Assay Kit (Invitrogen, Carlsbad, CA, USA) on a fluorescence microplate reader (Tecan, Männedorf, Switzerland) according to the manufacturer’s protocol. The values were normalized to the total amount of protein in the samples. Ten pea aphids from each group were evaluated in the assays.

### Hemocyte phagocytosis assays

Hemolymph collection and hemocyte treatment were performed as previously described [15] with some modifications. The aphid legs were gently removed with tweezers and drops of hemolymph were mixed with a drop (5 μL per aphid) of Grace’s medium (Sigma) containing 1 μM phenylthiourea (Sigma) and 10% (vol/vol) heat-inactivated fetal bovine serum (FBS) (Gibco, Grand Island, NY, USA). Hemolymph from 20 aphids per test group was collected and mixed well with 2 μL of 1 mg/mL *E*. *coli* (K-12) or *Staphylococcus aureus* Alexa Fluor 594 BioParticles (Invitrogen). Subsequent operations were performed in the dark. The prepared samples were transferred to tissue culture-treated round coverslips (8 mm diameter) in a 48-well cell culture plate (Invitrogen). The hemocytes settled and were allowed to adhere for 1 h at room temperature. The coverslips were then washed twice with hemolymph collection medium and the washed hemocytes were fixed for 10 min with 4% paraformaldehyde in phosphate-buffered saline (PBS, pH 7.4) and washed three times (10 min each) with PBS. To stain the F-actin, the hemocytes were permeabilized with 0.1% Triton-100 in PBS for 10 min and washed twice with PBS. The permeabilized hemocytes were incubated in the dark with SF488 Phalloidin (Solarbio, Beijing, China) diluted by 1:200 in PBS for 1 h. After washing three times with PBS, the coverslips were mounted on slides using anti-fading reagent (Solarbio) and observed under a laser scanning confocal microscope (FV3000, Olympus, Tokyo, Japan). The fluorescence intensities in phagocytosing hemocytes were calculated with ImageJ software (NIH, Bethesda, MD, USA). The phagocytic index was represented as the capacity of hemocytes according to a previous description: Fraction of hemocytes phagocytosing (f) = *number of hemocytes in fluorescence positive gate*/*total number of hemocytes*. Phagocytic index (PI) = [*mean fluorescence intensity of hemocytes in fluorescence positive gate*] × f [80].

### miRNA target computational prediction and reporter plasmid construction

Three different miRNA target computational prediction programs, RNAhybrid, miRANda and TargetScan, were used to predict putative targets of the miRNAs. After prediction, the reporter plasmid was constructed as previously described [81] with some modifications. The open reading frame of green fluorescent protein (GFP) and 3′ UTR of JNK containing putative target sites (739 bp) were amplified by PCR using *Pfu* high-fidelity thermostable DNA polymerase and primer pairs (*GFP* F/R and *JNK*-3′UTR F/R) (Table 1). The resulting PCR products were purified using a Gel Extraction Kit (Omega) and then used as templates to amplify the GFP-JNK 3′UTR DNA fragment by overlapping PCR using *Pfu* high-fidelity thermostable DNA polymerase and primer pairs (*GFP* F and *JNK*-3′UTR R) (Table 1). The product was purified and inserted into the KpnI and EcoRI sites of the PAC-5.1/V5-HisB plasmid (Invitrogen) to crate the PAC-5.1/V5-HisB-GFP-JNK 3′UTR reporter plasmid.

### Cell culture and reporter gene assay

*Drosophila* S2 cells (Invitrogen) were grown in Scheider’s *Drosophila* medium (Sigma) containing 10% FBS at 28°C in a humidified incubator. Next, 400 ng (58 μL) of the PAC-5.1/V5-HisB-GFP-JNK 3′UTR reporter plasmid was co-transfected into 500 μL S2 cells (1 × 10^5^ cells/500 μL per well in a 24-well cell culture plate) with 4.5 nmol (2 μL) miRNA mimic, mimic-NC (negative control), and mimic-mut containing a 6-nucleotide mutation within the seed region (5′-GGACGGA-3′ mutated as 5′-GACAUUC-3′) of the miRNA mimic using 3 μL of Attractene Transfection Reagent (Qiagen). Mock and reporter plasmid transfections were also performed as negative and positive control groups for GFP reporter analysis. Cells were collected and lysed at 54 h after transfection. Each sample was mixed with SDS loading buffer and boiled at 100°C for 5 min. After centrifugation at 10,000 ×*g* for 3 min, the samples were separated by SDS-PAGE, electro-transferred onto a polyvinylidene fluoride membrane, and subjected to immunoblot analysis using 1:5,000 diluted GFP antibody (GenScript, Nanjing, China) as the primary antibody. Expression of the GFP reporter gene was visualized using a Western-Blotting Detection Kit (Advansta, San Jose, CA, USA) on a chemiluminescent imaging system (ChemiScope Mini2950, Clinx, China). Three biological replicates were performed, and 1:5,000 diluted β-actin antibody (GenScript, Nanjing, China) was used as a loading control.

### Statistical analysis

All data were plotted using GraphPad Prism 5.0 (GraphPad, Inc., La Jolla, CA, USA). The log-rank (Mantel-Cox) test was used to analyze the pea aphids’ survival curves. Student’s *t* test was used to determine other statistical values, which were presented as the mean ± SEM.

## Supporting information

S1 Fig Effect of silencing of *JNK* on the mRNA levels of *Puc* in pea aphids with the aphids injected with dsGFP (as control group). The expressions of *Puc* were normalized with *rpl7* of pea aphid, and the relative expression of dsJNK injected groups was compared to the expression of the control groups at each time point. The values shown are the mean (±SEM) of three independent experiments and the statistical differences between the compared groups were denoted with asterisks. P values were determined by Student’s *t* test. *P<0.05; **P<0.01.
(TIF)

S2 Fig Eiger responds to bacterial infection, but Eiger does not regulate JNK pathway. **(A)** Relative expression levels of *Eiger* in the pea aphids after *M*. *luteus* (M.l) and bacteria *P*. *aeruginosa* (P.a) infections with the aphids injected with sterile 0.85% as control groups. The relative expressions in the infection groups were compared to the expressions in the control groups at each time point. **(B)** Efficiency of RNA interference-mediated knockdown of the pea aphid *Eiger*. The relative expression of the dsEiger injected group was compared to the expression of the dsGFP group at each time point. **(C)** Effect of silencing of *Eiger* on the mRNA levels of *JNK* and *Puc* in pea aphids with the aphids injected with dsGFP (as control group). For **(A-C)**, the expressions of *Eiger*, *JNK* and *Puc* were normalized with *rpl7* of pea aphid. The values shown are the mean (±SEM) of three independent experiments and the statistical differences between the compared groups were denoted with asterisks. P values were determined by Student’s *t* test. *P<0.05; **P<0.01; ***P<0.001.
(TIF)

S3 Fig Genes in the Eiger-Wengen-JNK pathway and IMD-JNK pathways in hemipteran insects. White frames indicate absent, gray frames indicate equivocal or unknown, color frames indicate present. Red: genes present in *Acyrthosiphon pisum*; blue: genes present in *Nilaparvata lugens*; green: genes present in *Cimex lectularius*; yellow: genes present in *Bemisia tabaci*; purple: genes present in *Diaphorina citri*. The numbers under the color frames are the corresponding gene IDs.
(TIF)

S4 Fig **(A)** Cloning strategy of the 739 bp DNA fragment containing target binding sites predicted of JNK 3′UTR under the GFP open reading frame into pAc-V5-HisB vector. The bases highlighted by red color were the seed region predicted. **(B)** The 813 bp, 739 bp and 1532 bp DNA fragments on the nucleic acid electrophoresis were the GFP open reading frame, the fragment containing target binding sites predicted of JNK 3′UTR and the overlap DNA fragment of the GFP ORF-JNK 3′UTR cloned into pAc-V5-HisB vector.
(TIF)

S5 Fig miR-184 is highly conserved among animal species. **(A)** The miR-184s of insects (*Acyrthosiphon pisum*, *Drosophila melanogaster*, *Aedes aegypti*, *Apis mellifera*, *Bombyx mori* and *Tribolium castaneum*), mammals (*Mus musculus* and *Homo sapiens*), fish (*Danio rerio*) and amphibian (*Xenopus laevis*) were retrieved from miRBase (http://www.mirbase.org/). **(B)** Sequence comparison of miR-184s listed in **(A)** using ClustalX2.
(TIF)

S1 File AP-1 DNA binding sites predicted in the sequences of *OXR1*, *PPO1*, *PPO2*, *TepIII-1*, *TepIII-2* and *YKT6*. (DOCX)

S2 File Prediction results of hybridization between JNK and miRNA-184 in insects, zebrafish, frog, mouse, and human using the RNAhybrid program. (DOCX)
