# Supplementary figures and images for: JNK pathway plays a key role in the immune system of the pea aphid and is regulated by microRNA-184
Source: PLoS Pathog. 2020 Jun 25;16(6):e1008627. doi: 10.1371/journal.ppat.1008627 (PMC7343183; doi:10.1371/journal.ppat.1008627)

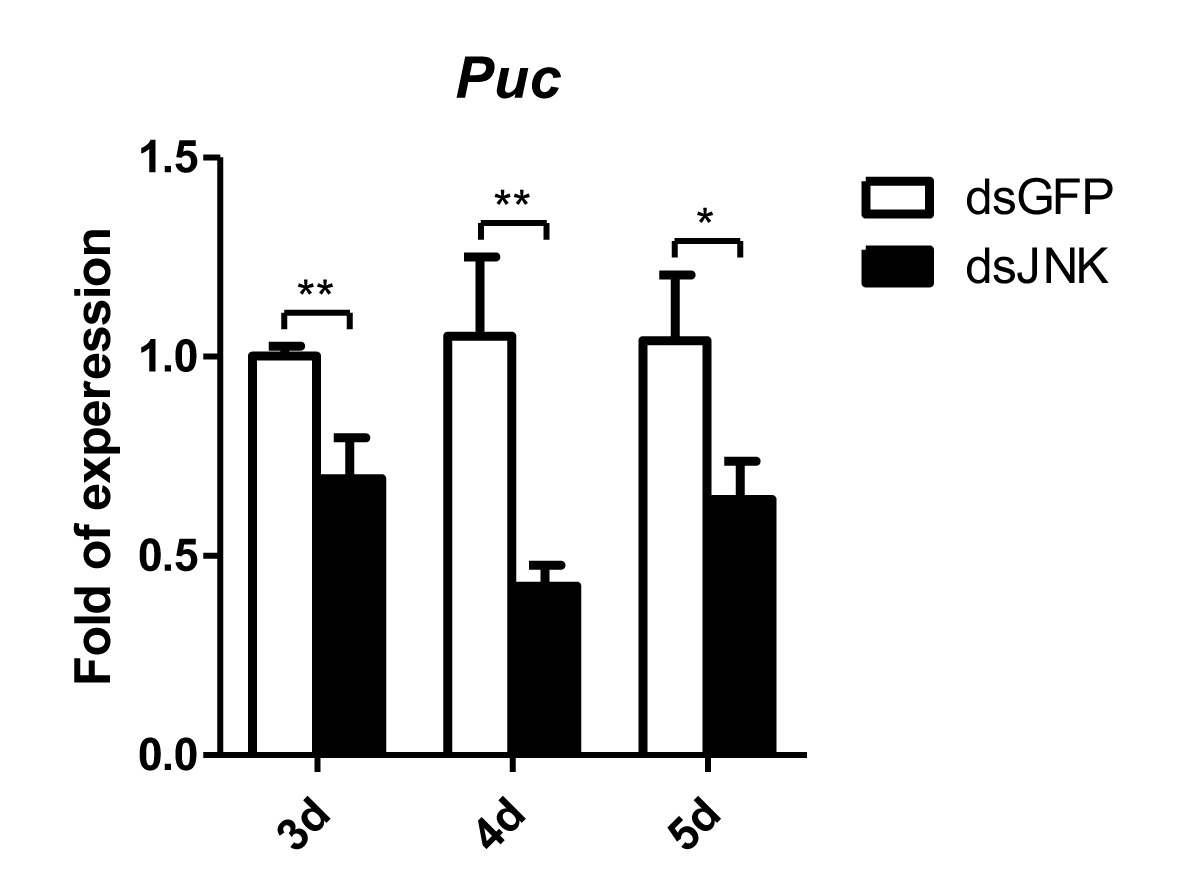

Supplement: S1 Fig — The expressions of Puc were normalized with rpl7 of pea aphid, and the relative expression of dsJNK injected groups was compared to the expression of the control groups at each time point. The values shown are the mean (±SEM) of three independent experiments and the statistical differences between the compared groups were denoted with asterisks. P values were determined by Student’s t test. *P<0.05; **P<0.01. (TIF) [file ppat.1008627.s001.tif]

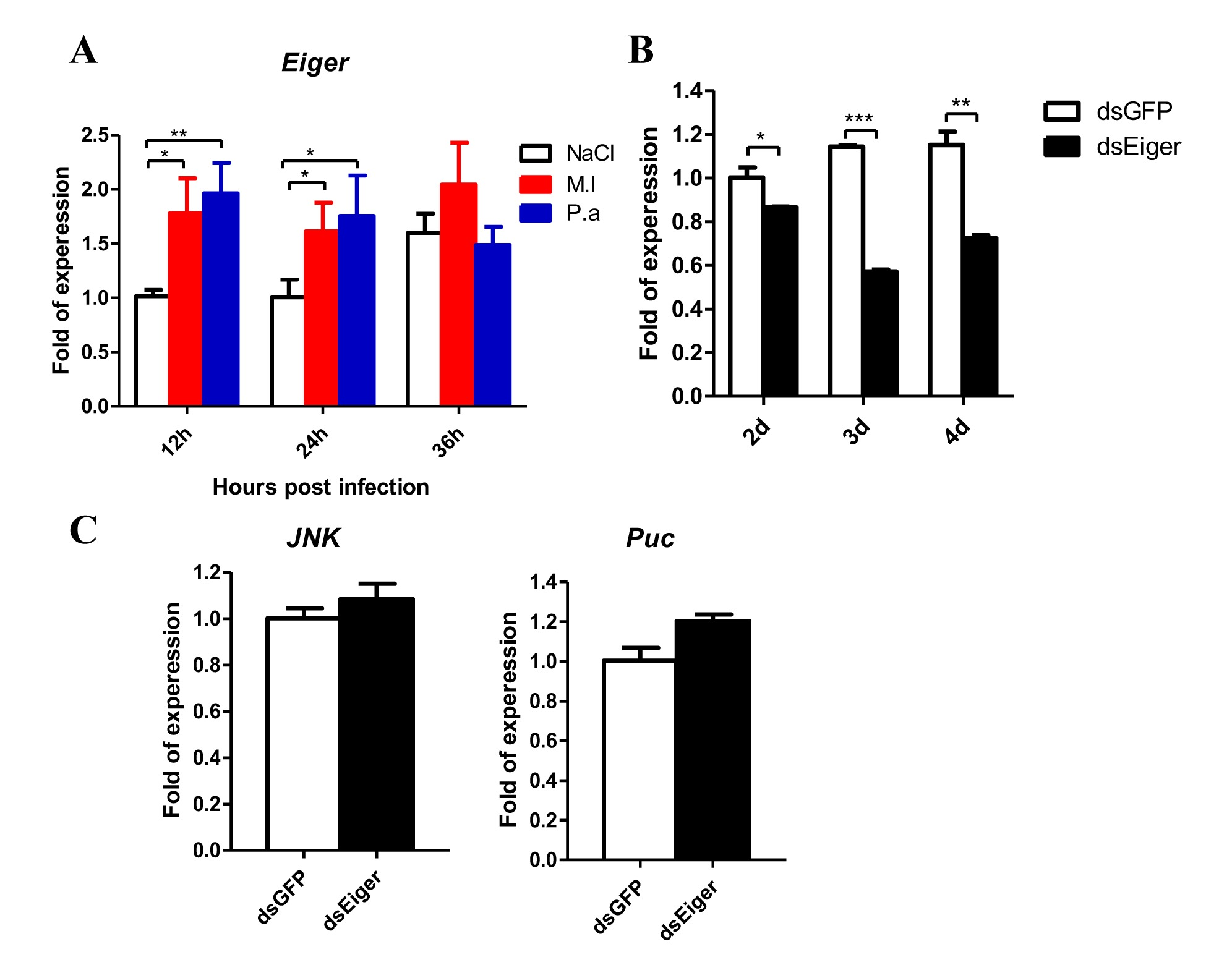

Supplement: S2 Fig — (A) Relative expression levels of Eiger in the pea aphids after M. luteus (M.l) and bacteria P. aeruginosa (P.a) infections with the aphids injected with sterile 0.85% as control groups. The relative expressions in the infection groups were compared to the expressions in the control groups at each time point. (B) Efficiency of RNA interference-mediated knockdown of the pea aphid Eiger. The relative expression of the dsEiger injected group was compared to the expression of the dsGFP group at each time point. (C) Effect of silencing of Eiger on the mRNA levels of JNK and Puc in pea aphids with the aphids injected with dsGFP (as control group). For (A-C), the expressions of Eiger, JNK and Puc were normalized with rpl7 of pea aphid. The values shown are the mean (±SEM) of three independent experiments and the statistical differences between the compared groups were denoted with asterisks. P values were determined by Student’s t test. *P<0.05; **P<0.01; ***P<0.001. (TIF) [file ppat.1008627.s002.tif]

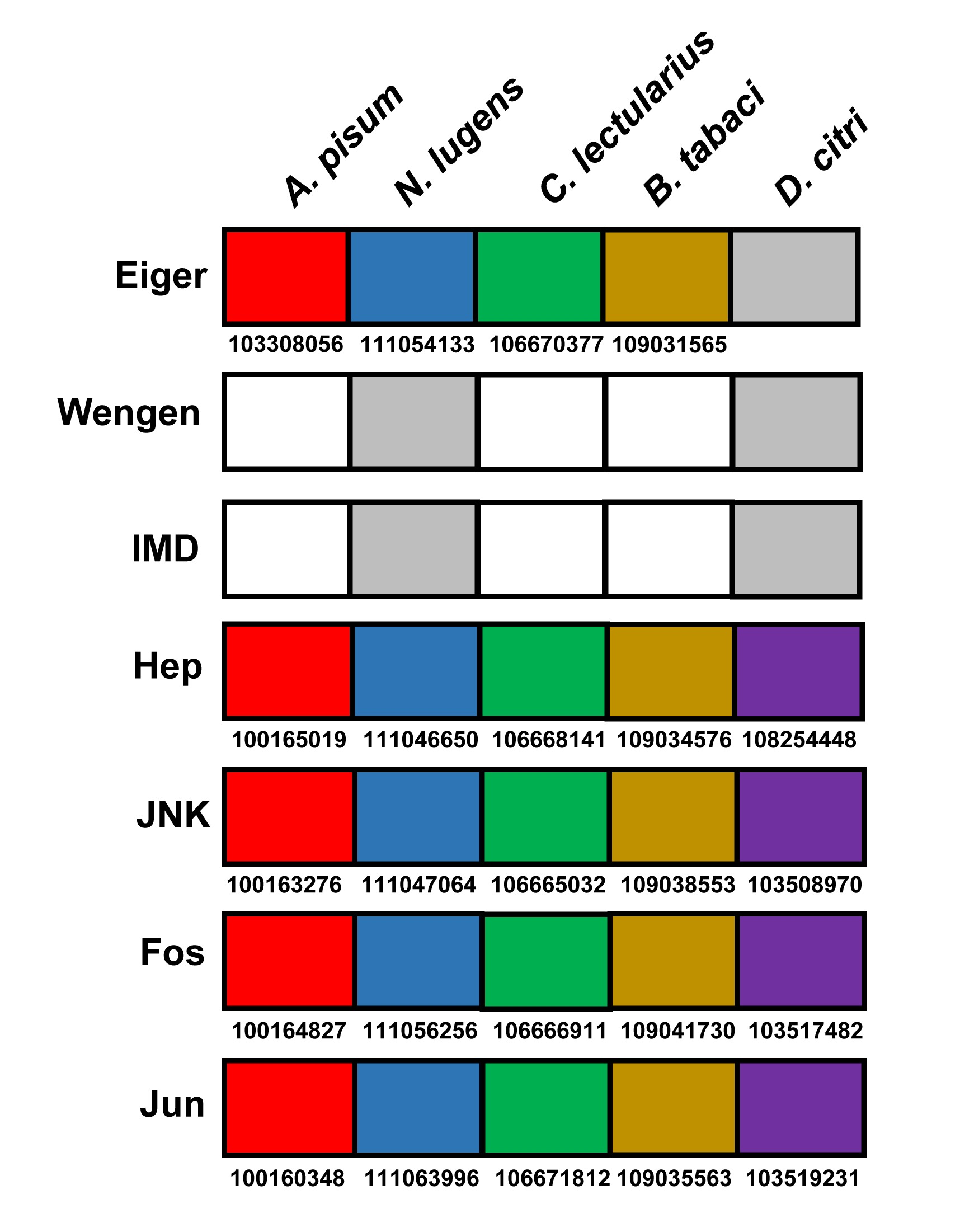

Supplement: S3 Fig — White frames indicate absent, gray frames indicate equivocal or unknown, color frames indicate present. Red: genes present in Acyrthosiphon pisum; blue: genes present in Nilaparvata lugens; green: genes present in Cimex lectularius; yellow: genes present in Bemisia tabaci; purple: genes present in Diaphorina citri. The numbers under the color frames are the corresponding gene IDs. (TIF) [file ppat.1008627.s003.tif]

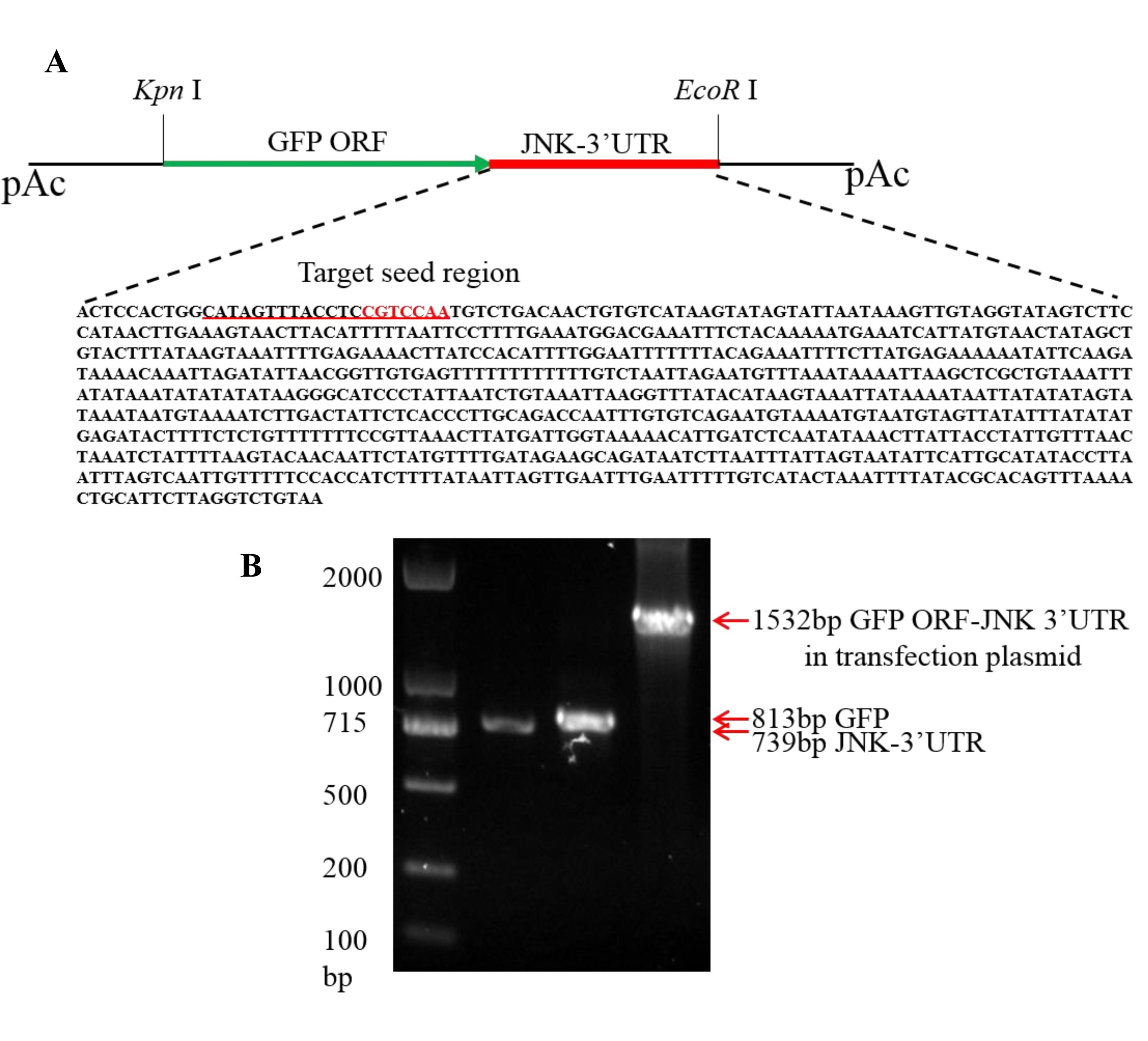

Supplement: S4 Fig — (A) Cloning strategy of the 739 bp DNA fragment containing target binding sites predicted of JNK 3′UTR under the GFP open reading frame into pAc-V5-HisB vector. The bases highlighted by red color were the seed region predicted. (B) The 813 bp, 739 bp and 1532 bp DNA fragments on the nucleic acid electrophoresis were the GFP open reading frame, the fragment containing target binding sites predicted of JNK 3′UTR and the overlap DNA fragment of the GFP ORF-JNK 3′UTR cloned into pAc-V5-HisB vector. (TIF) [file ppat.1008627.s004.tif]

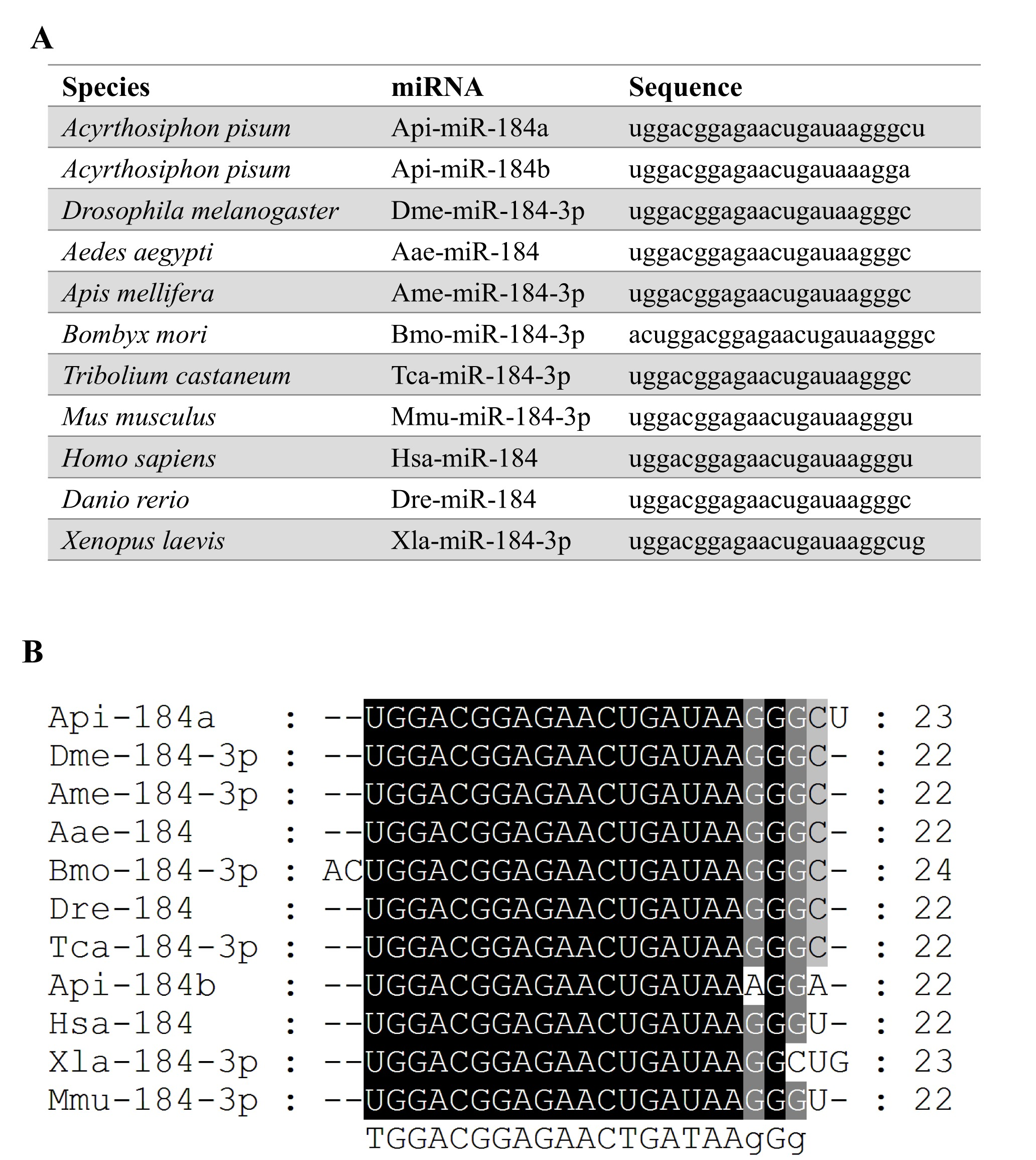

Supplement: S5 Fig — (A) The miR-184s of insects (Acyrthosiphon pisum, Drosophila melanogaster, Aedes aegypti, Apis mellifera, Bombyx mori and Tribolium castaneum), mammals (Mus musculus and Homo sapiens), fish (Danio rerio) and amphibian (Xenopus laevis) were retrieved from miRBase (http://www.mirbase.org/). (B) Sequence comparison of miR-184s listed in (A) using ClustalX2. (TIF) [file ppat.1008627.s005.tif]
