# Supplementary material for: JNK pathway plays a key role in the immune system of the pea aphid and is regulated by microRNA-184
Source: PLoS Pathog. 2020 Jun 25;16(6):e1008627. doi: 10.1371/journal.ppat.1008627 (PMC7343183; doi:10.1371/journal.ppat.1008627)
Supplement: S1 File — (DOCX) [file ppat.1008627.s006.docx]

>NW_021764024.1:c7581-5582 Acyrthosiphon pisum isolate AL4f unplaced genomic scaffold, pea_aphid_22Mar2018_4r6ur Scaffold_15231;HRSCAF=15887, whole genome shotgun sequence (OXR1)

TTCTTTTATTATTATAATTTTTTTTTTTTTTTAAGTATTTTAGACGGTATTATTTATATATATATATAATTAAAAATTTAAATTAAAAAACATTATATTATAATTGTTACTGTTACGCAAGTGATTTATTTTGATTTTGACGTGAAAAATAAAGTTAATGTAACGTTGAAAAAACCGTCTCATTCTTACCCATTTCCTGTCTCGTCCACTCCATCTATTATAATTATTATTATTGTAACTTAATCGAATGCTTAACGTGTTTGATTTTAAGAGTCGATGTCAGCTGAAGCGTTGGAATAGGTTGACACTTTTAAACTGTTTACCTACGTCGGGAATTGTACAGACGGGCTACTACCATCTGTGGATACTGGAGACTACGTATGGATGATAGGCAACGCTGTCTGACCTGCCAAGTTTCCGGTCCCTCATATACACATTAAATATTTTTCATTGTCTTTTATTAATCCCACTGAATATACGTATTCAATGATTATGAGTGTTATATGACTTATTATAACACTGTTATCGGGTCATTACATCACAATAATGTCCGACGGGAATTTTACTTGTATAATCTATTTTTATACGCTATCCAAACAATATTTTGTAGAAAAATATAGCCGACAGGAGCCAGGAAATGTGGAGCAAAGATTTCACATATTATTATAGGCAGTGGCATAATTAGAATTTGGTTGGGGGGGGGGGATAATAGGTTTTTAGCAAACATGAATGGTTATTACCAATACTTTGTTCAAAATGTTAGCAATAAAAAAGTATTTTTTTAGGGGGGGATCTACCCACTCATCCGCCCCTTAATTACGCCCCTGATTATAGGTATTATAAATTGGCCCTTCAGTAAAATTACGTACCTCGAGTTGGCCCTACCAGGCGTAAAATCCTGGCGCCACCTGACCACTGCGCCGCGCTTAATAACACCTTCAACAGCATTGGGCCATCATACACAGCAACAGGTACCAATAAGAGACGATACCCGATCACGTCATAAGCGAGGACCAATTAAAGACAACAATGGGTGGTGTCCTGATTAGATAACGGTTAGACACATGAGTGGTGCAGCGCGAGGGTATCTTAACAATGGATAAGACCATGACCATGGTATATAGAAAGGTGAGGATCACCAATTCGATTATAACACTTGCAGCTCCACATGAATAAGAAATTACTCGGAGAATCTGACGGAAACGAAAAAACAACATTAACAACTATGGTCACCGGTGTTGAATAAGGCCGAACGTACCTAAATATCATATTATGTATGGCACGTCCTATATAATCTACATAATAAATTGCACACATCTTTATAAGATTTGAGGATATATGTGACATTATTATTATTTGTATAGCTACTCATTTTTGTAGTCACTATATATTATAGACTATTTAGTTATCGTAACTTTTATCCACTAGTCATGCTATACATTTTTGTCTGGAATCCCGATAACCATGACGTTGCAGTTGAACAGTTATAATTCATAAATCACTATATAGACTACTAAGAGGTCCATGTACACTCTGGTGCATGTGAAATGTGATAAATAAGAATAGCGTTTTCAAAGTATAATTAATTATACCTAATAATATTACATATTACATTGTTTAATAGTTACAAAATATAGGAAGTATAGAACTTATAATGCAGAATTTATGAAATCGACTAAAAATAATTTTAACATTTAAAAAACACAATTTTAATGCACATAATATGTATTAAATATAGAACATACAGTAAATACAAAAATTACAAAATACCAATATATTAATAATAATTGATAAATACTACATTTTTGTACCTATATATAGGCGTACAGTGTGTTTAACGTTAAGGGTAGGTAAAACGAAACAAATTATACTTTTGAATTATACAACTCCCAAAATGTTCAGTTTAAATTTGAATTACATTTTTTTTTTTCAAACGACTAACTATTAAAGTTTTTATATTATACCTTAAAAATAGTATGAATAAAACAATTGAGGATTGA

>NC_042493.1:c88792234-88790235 Acyrthosiphon pisum isolate AL4f chromosome X, pea_aphid_22Mar2018_4r6ur, whole genome shotgun sequence (PPO1)

TACTGTGTATAATTTTATTTATACAAATTTATTTGTATACCATAATATTATAATATGTTCCTATTCTTTCTATTAATTAAAAATGCGATAAAACAATGAAGTTTTTTTAGTTATCTTATTATCTGTATGAAACTTGGTTTAGTAAGTTAGAGTACAACAAAAGAATTAAAAATATTTTATAAATTGATTATGTATATTGTGTTTTATAAAAAATAATTTGTTTATTTTTTGGGAGATGAAGCTTAGTTGGAAATCGGGTACAATTACTGGAACAGAGGGGTGGGATCGCTAATAACTTTAATTATAAAAAAAACACTGTTAACATTTTGAAACAAATAATACTATAAAATGCATAAGTTCTTTTTTTTATTTTGTTGGTGTTATAAATGGATAGATTCCACAGGATGTGAAATTATTTTGTAGAAATATATAAATTCATACAATTTTTAATTTAAATACCTATGCTGGGAATTTAATAAAAGGATCCTCATAAGTTGTTGTATTTTAATAAAAAAAAAAAAACCAAGGTGTAAAGCCACATTGAATAATATTTGAACGAAAAATATTACAATTTTAATCAAATTATATTAGTTAATTTGTTATTTTGTTAATTTGTTATTTAAAATAAAATATTAATTATATAGACTCGAAATTTCCCACGATTACTTATGTTATAATTTACTTTACATAAAGAAATGTTCAAAATATTTATACTAATTTTAAGCTATTTATAGGTATTACTTGACATTTTTGAATTCGTTTACTATTTTTCAACTTTTCATGACTTAAACATGAACAGTGTTCATGATAAGTTCTGCTTACAGCAATTTAAATTATCGAAAAGTCATGTCCACAGTTTTATTTTTTCAAATTTAAATTGTCTGTCCAATTCACTCAGAAGTCAGAATCTGAGAATCAATTTTTTGTATCCAATAATTTATCATTGAAGTCAAACCTATTATGCAGCAGAAGCGAGATCTCCGATTTTGAAAAATGTAGTCTTCAAATAATAGCAGGTGGTTCAGTGGTTCAGCCATTATGGTTACACAAGCAGTTCATGTAAAATTAGTAGTTTTTTTTGCTCAAAATATTTACCATTTTTACTAAGAAAATATTCTAAATATAACTTTATTATGAATATTTTGGGCAATTTTCATAATTGAAAATATTACAAATTATTTTTCTATTGCGTTTTTTAGTTGTGCTTACAAGACTTTTTTTGAGTGGTCTAAGTCCAACCAATGATTAGTCTTAATATAATTCCTTCTAGATACTTAATACCTATATGTACAGTATTATTTTAGTAAAACTTAAATTCTTCTGAAGGATTGAGGGTAATTATTATTAGGTACTTTTTATATTACTATATAAAATATTGTACACCACTCAAGTTTTCATACACTTAACGTTGATCATTGATATAGAATATAAATATAATTATTTTTTCTGACTTGGTTATCTCTGTATTTTAGTTTATAACAAACGATTATTTATTATTATTATTATTTTGACAATTTATTGTTTAAGCTGCATAAGTTATGAAAAGTACAACATTAGACTTTCTTATATTCAAAAAGAAAAATGAATTTATTATATTATATAAGAGTTTTATTTATGTACATCAGTAGTTTATAAGTGAGTATTTGAAATAGTTTCGTACTAATATTCCGGTTTTAAAACTTTGTTTTGTAAAGTTTCTGTGACCATCAGACCATGTGTTATAATTATCATACTTGAAATGTATTGTTTACTTAAAAGTTAAAGATAAAATAATTTTGTTGATGAAAAATTATCTAAATACAAATTTAAAGAATGGAAATTTATAGTAGTTATTGGTATTTGCAACAGACTCGTGCATTTTATATTATATAGGAAATAATCTATATTAGGATACTAACATTATTTTAGTGTAGGAAATAAACAACAATTCAAATTATTAACTGGAAGATATACATTAATATTAGAAATATAAATTATTAATCACATCGTGGCTTTTGGTA

>NC_042493.1:84414283-84416282 Acyrthosiphon pisum isolate AL4f chromosome X, pea_aphid_22Mar2018_4r6ur, whole genome shotgun sequence (PPO2)

AGTCAGCCATGGACCTGACCTAATACTGATTACTGAGTACTGACTAAAAATGTGTATACATTATATNNNNNNNNNNNNNNNNNNNNNNNNNNNNNNNNNNNNNNNNNNNNNNNNNNNNNNNNNNNNNNNNNNNNNNNNNNNNNNNNNNNNNNNNNNNNNNNNNNNNNNNNNNNNNNNNNNNNNNNNNNNNNNNNNNNNNNNNNNNNNNNNNNNNNNNNNNNNNNNNNNNNNNNNNNNNNNNNNNNNNNNNNNNNNNNNNNNNNNNNNNNNNNNNNNNNNNNNNNNNNNNNNNNNNNNNNNNNNNNNNNNNNNNNNNNNNNNNNNNNNNNNNNNNNNNNNNNNNNNNNNNNNNNNNNNNNNNNNNNNNNNNNNNNNNNNNNNNNNNNNNNNNNNNNNNNNNNNNNNNNNNNNNNNNNNNNNNNNNNNNNNNNNNNNNNNNNNNNNNNNNNNNNNNNNNNNNNNNNNNNNNNNNNNNNNNNNNNNNNNNNNNNNNNNNNNNNNNNNNNNNNNNNNNNNNNNNNNNNNNNNNNNNNNNNNNNNNNNNNNNNNNNNNNNNNNNNNNNNNNNNNNNNNNNNNNNNNNNNNNNNNNNNNNNNNNNNNNNNNNNNNNNNNNNNNNNNNNNNNNNNNNNNNNNNNNNNNNNNNNNNNNNNNNNNNNNNNNNNNNNNNNNNNNNNNNNNNNNNNNNNNNNNNNNNNNNNNNNNNNNNNNNNNNNNNNNNNNNNNNNNNNNNNNNNNNNNNNNNNNNNNNNNNNNNNNNNNNNNNNNATACATTAAACACAATTACTACCTATATAAAGTTGTTAATAGAAGTTTTTTTTTTATCATTTGGCCTGCCATAATAAAAGTGTCTAGTTTCGCCTATGAATATATATACCTATATATAAATATATAATATAATTAATATAATACAATTATAATTACAATTACAATTAAAATATCATAATATTACAATATTACAACTATTGCAATATATTTATAGTATGTAATTGTATTATTAAACAAGTTTAATTATTAAACATAAACATCGTATAAATGTATAAAATTAATACGACCGCATCGGTATCTTACACGCTATGCCGCAGATAACCGATTCCCACGCACTTAAAAACACAACATATCCGAAATAATAATACACAAATAATTAGAAAAATAACGAACTATTAGAAATCAACTATCCAACATATTAAAAACAAAGTTTGAAATAATATCTAAGAAATATAGTTTCTAGGTATTGGACTCCCCATAAAAATATTAAATTTCAATAAAAATTTGATAAACTTTATACAATAATAAATTACAGAAAAAACTTAGATACCAATAGTAGAAGTTACAAAAATTAAAAATTAGCATTTAATTACAATTTACAAACAGGTAAACCAAATAAAAATGATGTTGGTTTATTAATTGCACTGATTGAAACGTTGGTTTATTTCATAATTTTTATAGGCAGGTTCATATTAATTTGTTTATTTGGTCACCATAACTATAAATTATAATTGTATAATGCAAATAATATGTAGTAGGGCACCAAGTAATTTTAGTAACTAGGGCACCATTAGGTGTAGTTGCGCCACTGAATACAAATATGAAATAAGGAACAGGAAATTTCATTTTTTATTAGAAAATATTAAAAAGGCTTTAGAGCAGTGGTTCTCAACCTTTTTTGGCTCACGGCACACTTTTATACATCACAAGTTTTCACGGCTCACTCACGCACTTGATAACCATAACAACTTTGAAAACACGCGTTCTCACCACGCGCCTATAATACATGAATCCGGTATCGTCTTATCTGCACAATTAGTAACAGATTAGATACAATTAGTACAAAACTGTGCTACAGTTCTAAAAACTACGTCGTATTAATCTACACACTTATGATTTTATCTGTATAGATAAAACTGTTCAGTCGAATAGTACAGTTTTAACTGCACGTGTGAATTTTCTCACTAGTTATTTAAAATGTATTCACTTTATGATATAAATATTCCTTTGTTT

>NC_042493.1:22604728-22606727 Acyrthosiphon pisum isolate AL4f chromosome X, pea_aphid_22Mar2018_4r6ur, whole genome shotgun sequence (TepIII-1)

AATATATTACCCACTGGCTTCTATATGAATACGATCATCTATATATCATGTACCAACGAAGTAATTTCCATTTTTATTTAACATACCTATTGTTTAATGTTGCTGATTTAACTTTGATTTTTAAAACTGCATGTGTGTATTAATAAATAACATGTTGTAATTGTAAACAAGATAACTCATGGGCATCCACACGAGTTTTTAACACGGTGAGGCCTAATAATATTTTTTTGATTAAAAAAATATCGGTTGATTCAACAATAATAATCCTATATATTACAAATATAATATCAACAATGTTCCTTAATTATGGCAACAATAATAATTTGTACATAATATAATTATACTAATTATCAAAACAACAATTTTTTTTGATTGACTCAACAATATCAGAAGTTGCAATTGAAAATAAAAACCAAATTTTTGTATTTTACTATATTATGTATTTATGTCATATATTATTCACATATTATTGTATTTTTTAAATATTTTAAATATTAATTTTTACTATACATATACCTAAGCTACTAAATTGTCGATATAAAAGTAAGTTTAAAAATATATAAGAAAAAATTTAAACCTTTTTTAAATGTTTACAATAAGTTACGCCAATTCAAACAATTTATATATAAATAGTATTTATATATTGTTCACATTGTTAAGTTACAGTATGAGCTATTTATGAGAATTGTTGTTTTAAATTTTCAATCCTTAGCTATAAAAATTGAACATTTTATAATTTTTAAATATGTTTTAAAAATTTGTCGCAATACAATAATAAGAATAATAATTTAAAATAAGTTTCACGTTTAAAAAATTGTCAATACAAACATAAATTACTACAGTCTACAGTTATAATAATAATAATTTCAAATAGGTTATACTGTTTAAAAAATTGTCAATACAGACATAAATTATATTAAATAATAAAAAATAATAAAGATTGATAATTTGTTCAGTTAAATTAAGTAAAAATAATAAAAATTACCAATAACAATTTTTCCAACATGGACATACTACTCCCAAAACATAGTATAACATAGTTAAGCTCGTTTTTATATATTATTTTTAATTATTCAAAATGTACTAATTAGGTTACGATAGTCAGTGGCGTTTATAGAAATAATTTTTGGGGTGGGCTATAGGTAGTAGATAGAAACTAGCAAGTGATCTATTACTTAGAGGCTACTCTATATGTTTAACAGTTTCTATAAATATATTCGTATTTGTTATTTTTCAACAGGCTATGAAGTATTATAACTTATAACAGTACCTGATAAAATATATTGTTCTATTTAAAAAGACTGTAATTTCGGGGGGGGGCACCCCCAATATACGCCATTGACGATAGTATTCAACAGATAGAACACCCGGGCAGTTGGATTATTGTTCAAAATTTGTATTGAAATAGGTACGATTTTAAATGTGTTTTGTTTTAGATTTCTAAATTGTTTAAACGTACCTATACACTATATATTAAACCGTCCTTCCAAATATCTTAAATCATAGTCTAGGAAAGGAGACGTAAAATGTCAACTATACAAGAAATAAGAAGAAGGGACCCCTCTCCACACTTTAACACTGGTCAAGTTTACATTTAAATTTATTTAATATTTAAACCGTGGGAGATGGAACCTACTATATACCTAGTATTATATTATTATTTAACGGCGAACTAAGACGTGTCCGAGGACAACTTCGTCATAAGTGCTTATTTTTCAACCATAAATATGTTTATGTCTATTATTAATCGTATATGAACCAGTATATACATAGTATATACCTATATATCGACGATATACCACATTATATACACCTGTTCATGTATATAACACACACACAAACTCGTAGACGAAACGGAACTGGTTTGGATCGAGATCGTCCCCTCCACCCGTGTCTCGATCACTACTATACTACTACTACGCCACCACCTCCATCATCAGCACCACCGCCGTCACGTCCACCTACCTACCTCCCGAAAACACCGAAACCGTATAATGACT

>NW_001934300.1:c17410-15411 Acyrthosiphon pisum genomic contig, reference assembly (based on Acyr_1.0 SCAFFOLD5597), whole genome shotgun sequence (TepIII-2)

AATTTAATTTCTTTAGTACCTAATTACAGAGAGACTTCTAAGATGTGCATGCAGATTTATTCATACCACCCCAAACTACAATTCTGTATTACAAAATACTAAATAGATTGTATTAGTGCAAAATAAACCATTTTTAGAGAATTTATTTTAACAACTTAATCTTATAAAACAAGCGTTGTGTAAATTAATAAATGTACTAAATGGGTTCATTCCATTTAAGGTGTTCATCAATAATAATACCAAAATATTTTATACTTGACATTTTTTGTATAAGTACCTATACAATTATTATGGATGATGCGATCTATACATTTTTTTACATGGATTATGCTAATACCTTTATTTAAATGATTTTTATGAATAATAACAGATACAAACTCGGTTTTTTGTAAGTTAAGCTTAACCCTTAAATTGGCAAACGTAGAAAAAGAGACCTAAAAATTATAATGTAAGATGTTTCTGCCTAATTTGAGGCTGCTGAATCCAAAAATGTTGTCAATTTTTTTGTACAACTTAAAGTTTTTGACTTACAAGTTGTATCTTGAGGGATACATTGCCAAATGCATGCAATGATAGAGTTGTATCTGATAAGATACATTGCCAAGCTTAAGAACACAATTATTGTGCAGTTGAAATACATTTTTATAAAACATAAAATAATAAAAATTTATAAAACTTTTTATATTTGTATATAGAATATATAAAATAAATTATTTAACTTTTTACTTATACAAAATAAACATAATAATACTATCTTCATTAAATAACATGAATAAATCTATTGTTAATATGTAAATATAGAATTTATAAAATGAATCAAACAATTTTTAATCTAAGAACAAGATACAAATAAAATAAAATCACCCTTTTTAAATAATATGTTTTATAGGAACAAATGAAATAACATCAAATTTTGATATATTGTAATGAGACAAAACGGAGCGTCCAAATGCGTGGCGCGCTCTAGTTTGCACATTACCCTCAAGCAATATTGATCGCCGCCGTGCGATATCGATTACGCGACGGAGCGCAACTACAACTAGAAAATACAGATAGCGCAGGTCGGCATGGCAGTTTTATGAAAGCATGCCGCCCGCCGGAAAACATGAGTTTCCGAATGAAATATTAAAGCTAGAAAAAAGCCAAGCACATATTCGAAATCAGCAGAAAACGTACAATAAGATTAATATTATCAAATATCATAAACTAATACTGGAAAATCAGGAACACGTGAAACATCAAATAAAATTGTGCACGGGACCAGCGAGCTTCGAATAGCATAAATACAGGCCGCTGGCCGCCGTCCAGACAAGACCATCTTCCCCGCTGCGAGGAAAGAACAGTAAGCCGGACAATAAAAATATTGCCAAGAACGGATACTGATCCCACTCGACCAACAGCAACTTGCCCCAGGACTGAATAAGCCGGACGATACGCGTATCGTCAAGAACGGATATTCGTAAATAGTCTGACCTGGGGGTCGATTCTTGAATCTCGTTAGGAAAATCACTCATGAGACATTTTCCGTACTGTTATGTCGTACGACATCTGAAATGCCTATTCTTGAACTACACACGACATAATATGATTGTGAAACGAAATTATTTTGTCACACACGAAATATTTTTCACATGAACCAAGTTGGTTACTAACCCTATCATGATTATAATAAATAATAATTGTGATAACACCACCGGTATGGCGGTATTTTGATTAAATGATTGTTATCGATGTATCGTAATTCGTAGTATAGTATGGTTACTAAAGTTTTTATAATTCTATACCTAGTTTTATAAGTTTGTATATATGATGTAAACTTATACTTACTGTTAAACAGAGTTACAACTTACAAGTACAAGTTTATAAAGTAGGTACAATAATTTGTTTTTTTAATGGAAGACTCCAAAAAAATATATTGTTCAAAAACACAAAAAGAGATGCTCATCCAACTTCTGACAAAAGATCCACAATTGATTTCTGGGAAGTTTACAAGCACTTTC

>NC_042496.1:c8032215-8030216 Acyrthosiphon pisum isolate AL4f chromosome A3, pea_aphid_22Mar2018_4r6ur, whole genome shotgun sequence (YKT6)

TTTGTATGTCTTTATTCTTTAATTTAGGCAAATGATGGGCGTTTTATTTATTTATTCCTAAGCAAATATT

TAATTTATAGTTGATCAATACTAAAATGTTTTGATCTATTATGATATGTAATTGATAATTTATAAATACT

TATATTATATTGATGGAAATAATAGAACTATAATTATTGGATCATAATAATAAAATATTGTTATTATGAA

AAAATAGATTTTCAGATTAGTTCAATAATACATTAATACATCAATGTTTCCCTTCCCAAATGTATTAAAC

TGGTTAGATAATTAAACCAATATCTACTAATCAGTATTTGAAATAATTTTCAAATACAAAACAAAAAAAT

CAATTTAATTCTGAATCATTCATAATATATTATATTAATCGAACATTATCTTAAGTGTTGCATTAAGAAC

GTGTACAAGATACTTCATGTTCGTATTATATTTCGCTATAATATGTTATATTGGTAGGTACCGAAAACGT

ACTGTTGCGATCGTTGTCGAGTGTCGACGATAAACAGGCAGTTTCTCGGAGCAAAAAATATCTAGTTAAG

CTATTAATAGATGAAAACTAAAACCAATATAGAATTGTTCATAAATGATAAATATATATAATATTATTAT

TTGACAATGAATAAAGAATTAAAGGAATAAAAAAAGTATTCAATAATAAAAAAATGTACCATTTAAAATA

CCGTTCAAATACTTCAAAAATAAAAGTATTCAATACGGAAAAAAGTATTTGAATACTTCTACTCAAATAC

TTTACAACACTGCTACTAATTAAGTTATTCATTGAATTATTTTAAAAATATAACAGGTATGTACAACTAA

AAATATTTTTTAATTACTGTTGTTTGTTACTTCTAATTTCTAAATTTAAAATCTATATTATATTATATTA

AATACCTAGCCTAAATGGGTATATACCATATTACCATGCTATACATAGACTCGGAATAATATTACAGATT

TTGTAATTAATTATTTAAAGTAAATTAAATAAAATTAGTTTTGTTACATTTTCAATATCCGTTAAAATAG

TAGAGTTTTTTAATCACCCTCCACCCACTAAAAGTTATACGACACTGTATTAACTTCTACATAATATTAT

ATTTAAATTAAACAATAATCATCTCAGTTCCAACTTTCTTCTAGTTAAGAGAACGCTACACCCGCGTCTT

GCATGCGTTTTCTCTGTCTTACAATTAGTGCACAACATAACAAAAACTGTTTTTCGCGGGACAACATGTT

CCCTTTGTATTTATAGTAGAATTACCAAAATTCCACAACGCATAGGGGATATTCATCGTAGAGTTTTTAT

TATTTGGACAAAATGAATACAATTAAAGTTATCAGTTTGAGAACATTAGGTTTTTTTTTTTTTAATTATT

AACAATTATTTGTAAGTTCTATCAAAAAAACAGAAACGCTACGACACAGATTAAGTTCATCCCTATGCGC

CGTGGATTTTTGGTAATTCTACTATATATACAGAGGGAGCATAATTGTTCCACTAAAAACAGTTTTTGCT

ATGTTGTGCATTTGTAGGACGGAGACAATGCATGCGGTTGTAGCGTCCTCTTAAGTTAATAGAGCTTACT

ATTGTTTCATATATTTATATATGAAAATATAGGTACCTATCTAATATATAAAAATAGACGTTTGTTTGTT

CGTCCTCCATCGACTCAAAAACTACTGGACTGTTTTCAATAAAAGTTATATCAATCGATTCCTTGGGGTT

TGGGACTCACAGAGGGCTTGTAGCTTAGGTTTTCAATCCTTATAGGGTGGCTCATACATATAATGTTTTG

TTTTGGATCGTTATATAACATAAGTATACACAACGTGTAGCCGTGTAGGTAATCAGAATAAGAAGTTTTA

ATTTTGCATTTATTTTTATTTTTTTAGGGCAATTTTGGAGTTTTTGAACGCAACATATTTTGAATATTAA

TTTTAAGATTTTTACATGATTCTTATACAAATTATTAACA
