# Supplementary material for: JNK pathway plays a key role in the immune system of the pea aphid and is regulated by microRNA-184
Source: PLoS Pathog. 2020 Jun 25;16(6):e1008627. doi: 10.1371/journal.ppat.1008627 (PMC7343183; doi:10.1371/journal.ppat.1008627)
Supplement: S2 File — (DOCX) [file ppat.1008627.s007.docx]

**target: Drosophila melanogaster basket (NM_164900.3)**

length: 1535

miRNA : dme-miR-184-3p

length: 22

mfe: -23.3 kcal/mol

p-value: undefined

position 999

target 5' A CGUU GCAA ACAGCCAA A 3'

CCCU UAU CGGUU CCGUCCG

GGGA AUA GUCAA GGCAGGU

miRNA 3' C GA 5'

**target: Apis mellifera JNK (XM_016911128.2)**

length: 2467

miRNA : ame-miR-184

length: 22

mfe: -24.4 kcal/mol

p-value: undefined

position 2375

target 5' C ACGUUUCAGACG U UCCCAACAAAUAU GA U 3'

GCUCU UAUC GU UCUCCGU CCA

CGGGA AUAG CA AGAGGCA GGU

miRNA 3' U 5'

**target: Aedes aegypti JNK (XM_021848089.1)**

length: 5392

miRNA : aae-miR-184

length: 22

mfe: -24.8 kcal/mol

p-value: undefined

position 2286

target 5' A AA C 3'

GCC CA UUCUCCGUCU

CGG GU AAGAGGCAGG

miRNA 3' GAAUA C U 5'

**target: Bombyx mori JNK (NM_001109926.1)**

length: 2268

miRNA : bmo-miR-184-3p

length: 24

mfe: -27.3 kcal/mol

p-value: undefined

position 360

target 5' U CACG CUCCCG U 3'

GCCC CGG UCUCCG CCA

CGGG GUC AGAGGC GGU

miRNA 3' AAUA A A CA 5'

**target: Danio rerio mapk8a(NM_001110389.1)**

length: 1644

miRNA : dre-miR-184

length: 22

mfe: -24.2 kcal/mol

p-value: undefined

position 1201

target 5' A C CUCA U 3'

GCCCU AUC UCC UCCA

CGGGA UAG AGG AGGU

miRNA 3' A UCAAG C 5'

**target: Tribolium castaneum basket (XM_015982966.1)**

length: 1498

miRNA : tca-miR-184-3p

length: 22

mfe: -22.3 kcal/mol

p-value: undefined

position 332

target 5' A GU G UU A 3'

CUUUA UCAGU UU GUUCG

GGAAU AGUCA GA CAGGU

miRNA 3' CG A GG 5'

**target: Homo sapiens JNK1 (NM_001278547.1)**

length: 5854

miRNA : hsa-miR-184

length: 22

mfe: -25.1 kcal/mol

p-value: undefined

position 4354

target 5' G UUG U 3'

CCUUGUCA UUCUGUUC

GGAAUAGU GAGGCAGG

miRNA 3' UG CAA U 5'

**target: Xenopus laevis mapk8.L (NM_001086715.1)**

length: 3227

miRNA : xla-miR-184-3p

length: 23

mfe: -25.3 kcal/mol

p-value: undefined

position 3012

target 5' A AGUCCUU A G 3'

CCUUGUUAG UUCUC GUCUA

GGAAUAGUC AAGAG CAGGU

miRNA 3' GUC G 5'

**target: Mus musculus Mapk8 (NM_001310452.1)**

length: 5626

miRNA : mmu-miR-184-3p

length: 22

mfe: -26.7 kcal/mol

p-value: undefined

position 2286

target 5' G CC C 3'

GCCUUU CAGUUC CUGUUC

UGGGAA GUCAAG GGCAGG

miRNA 3' UA A U 5'
